# Supplementary figures and images for: PTX3 mediates PI3K/AKT/mTOR signaling to downregulate apoptosis and autophagy to attenuate myocardial injury in sepsis
Source: PeerJ. 2024 May 20;12:e17263. doi: 10.7717/peerj.17263 (PMC11114122; doi:10.7717/peerj.17263)

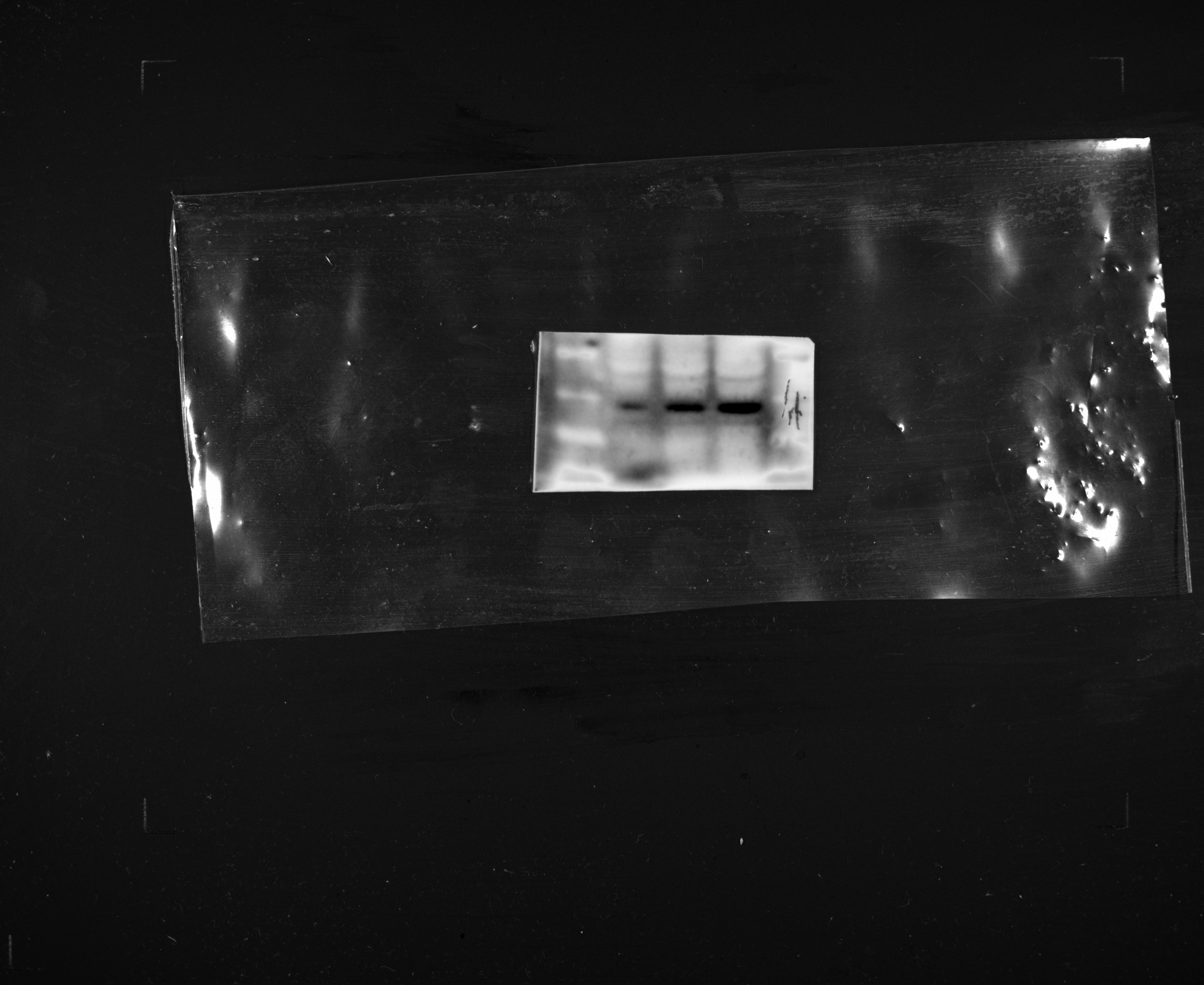

Supplement: Supplemental Information 3 [file peerj-12-17263-s003.zip › Figure 3 WB/001-merger[PTX3(1-1)0611].jpg]

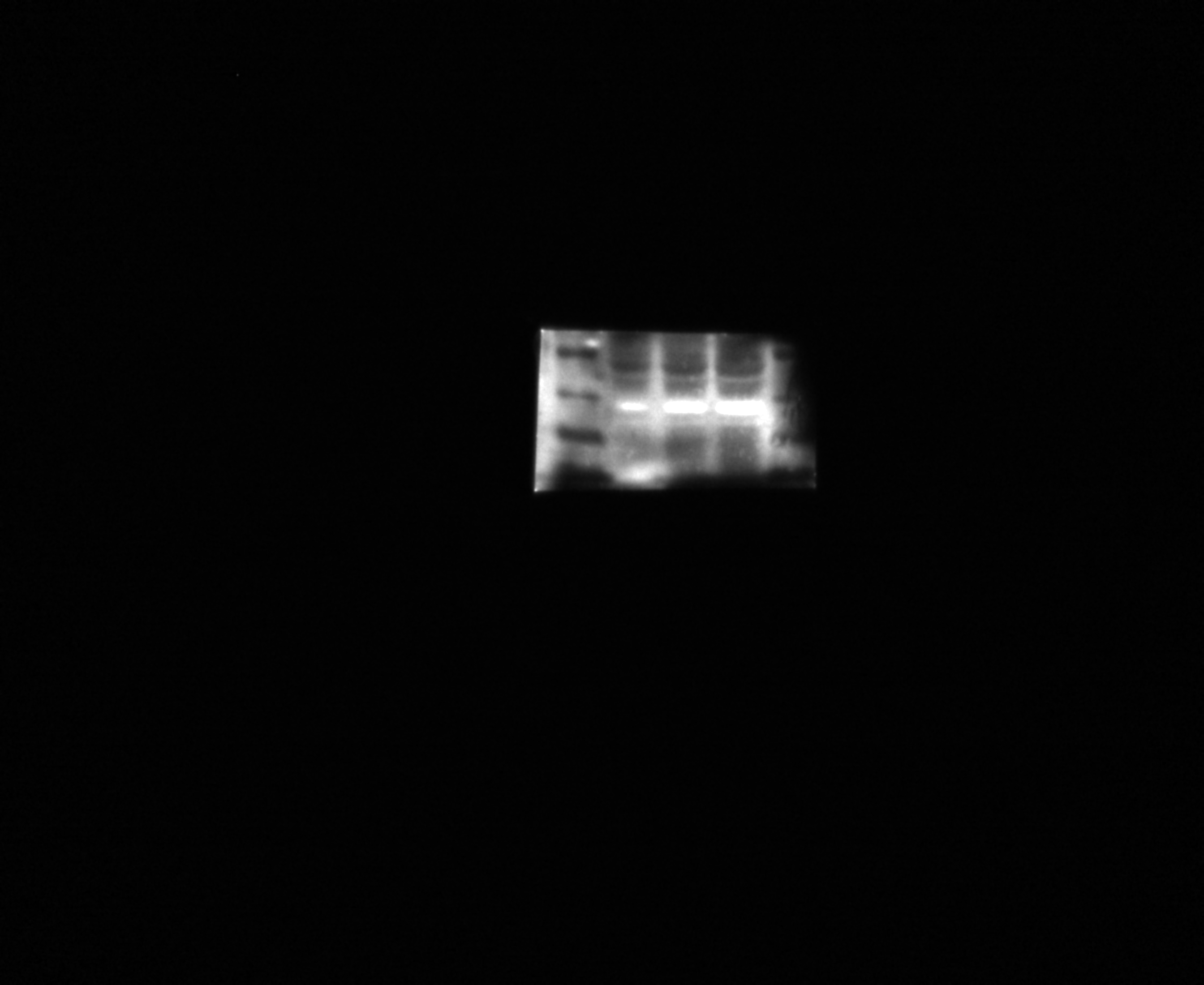

Supplement: Supplemental Information 3 [file peerj-12-17263-s003.zip › Figure 3 WB/001-shine[PTX3(1-1)0611]-raw[367,4958].tif]

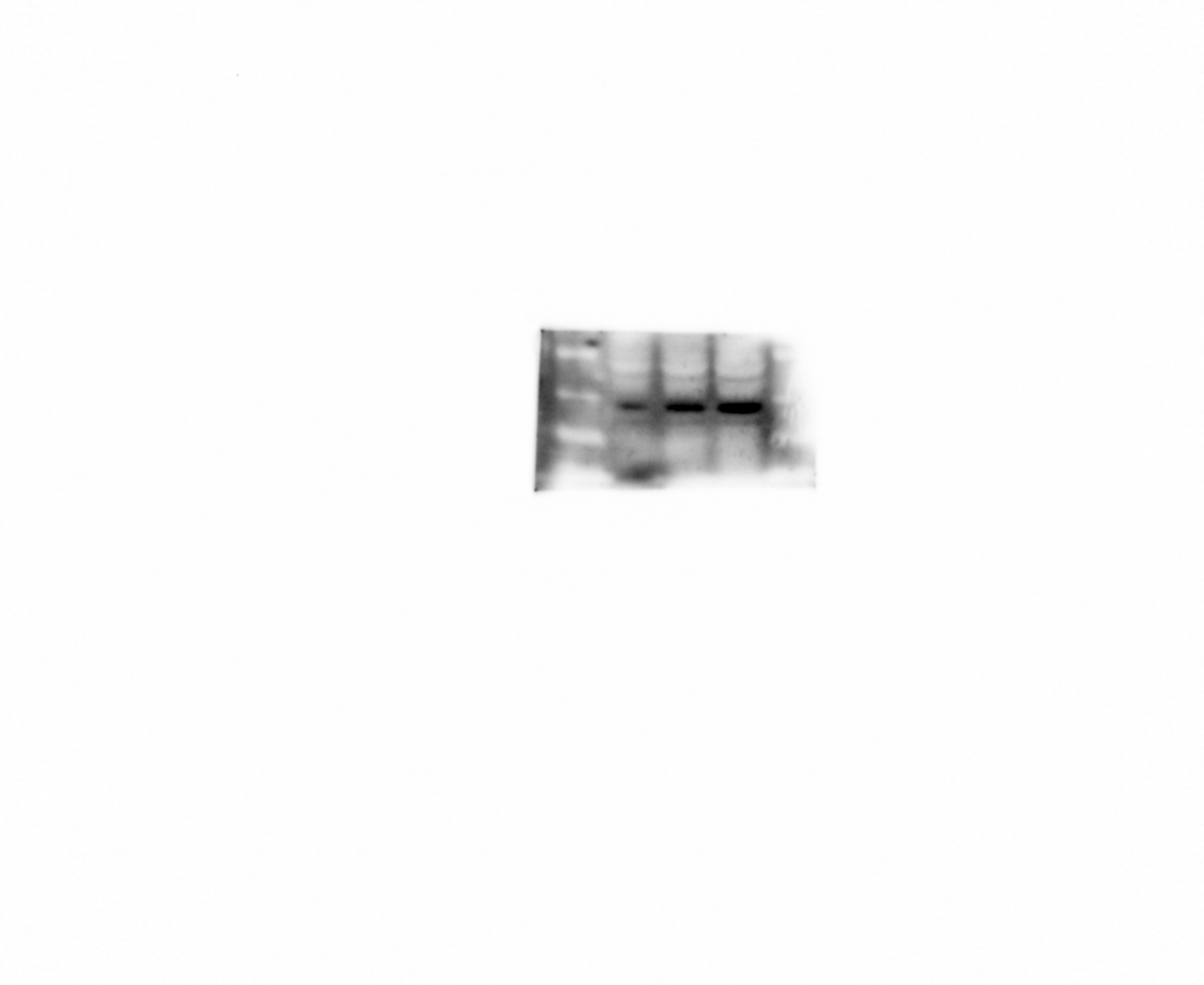

Supplement: Supplemental Information 3 [file peerj-12-17263-s003.zip › Figure 3 WB/001-shine[PTX3(1-1)0611].jpg]

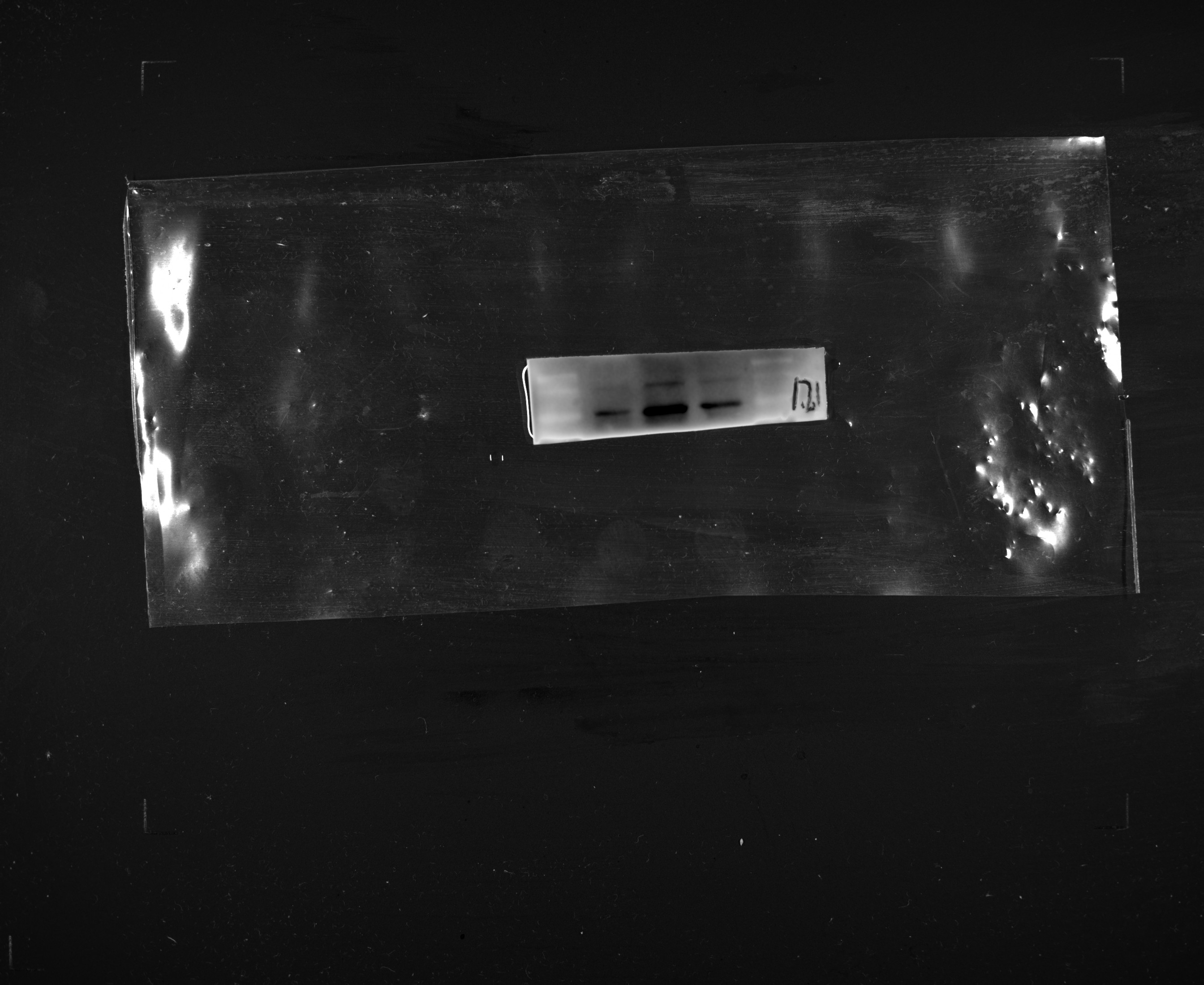

Supplement: Supplemental Information 3 [file peerj-12-17263-s003.zip › Figure 3 WB/002-merger[p-PI3K(1-2-1)0611].jpg]

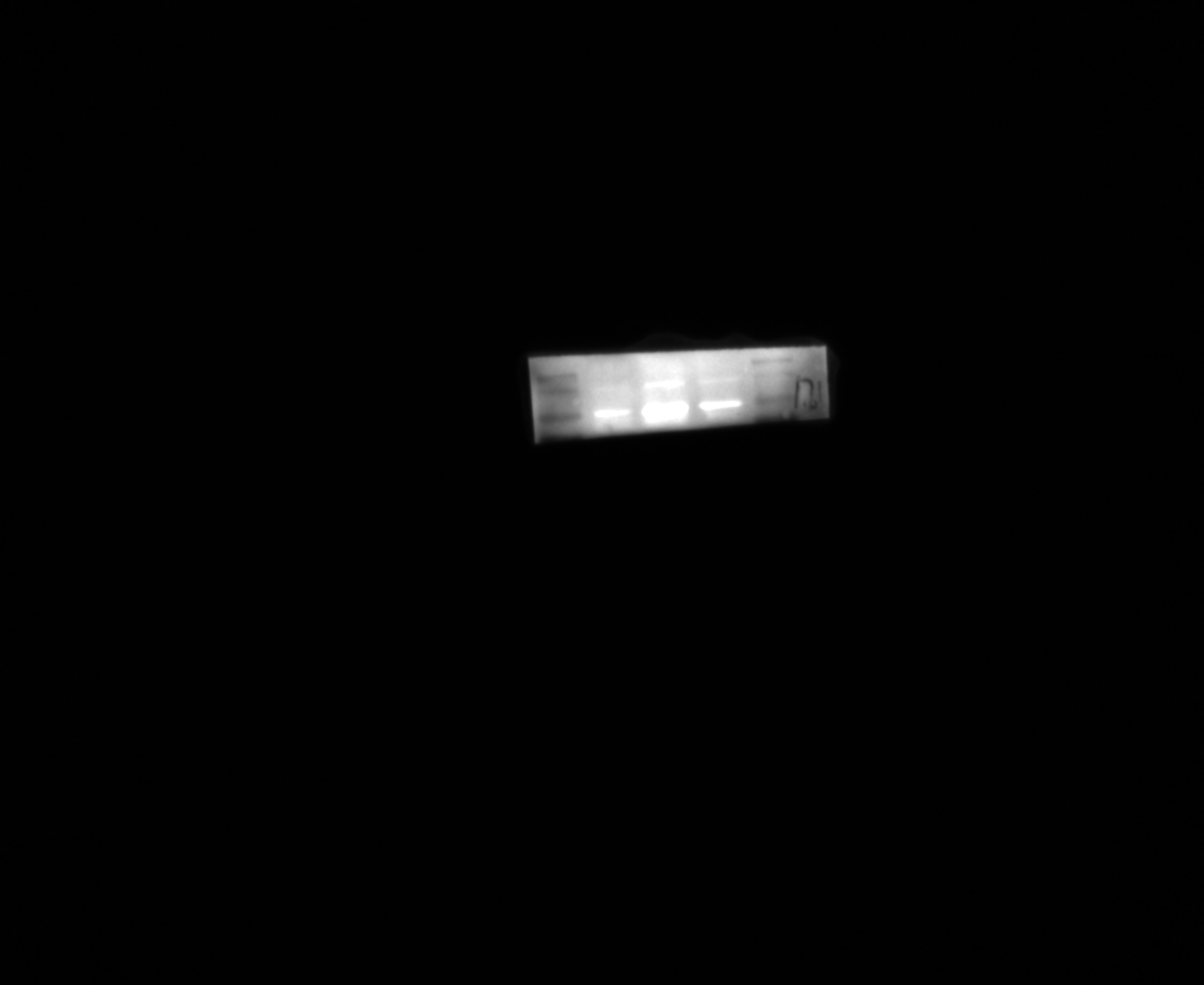

Supplement: Supplemental Information 3 [file peerj-12-17263-s003.zip › Figure 3 WB/002-shine[p-PI3K(1-2-1)0611]-raw[367,10890].tif]

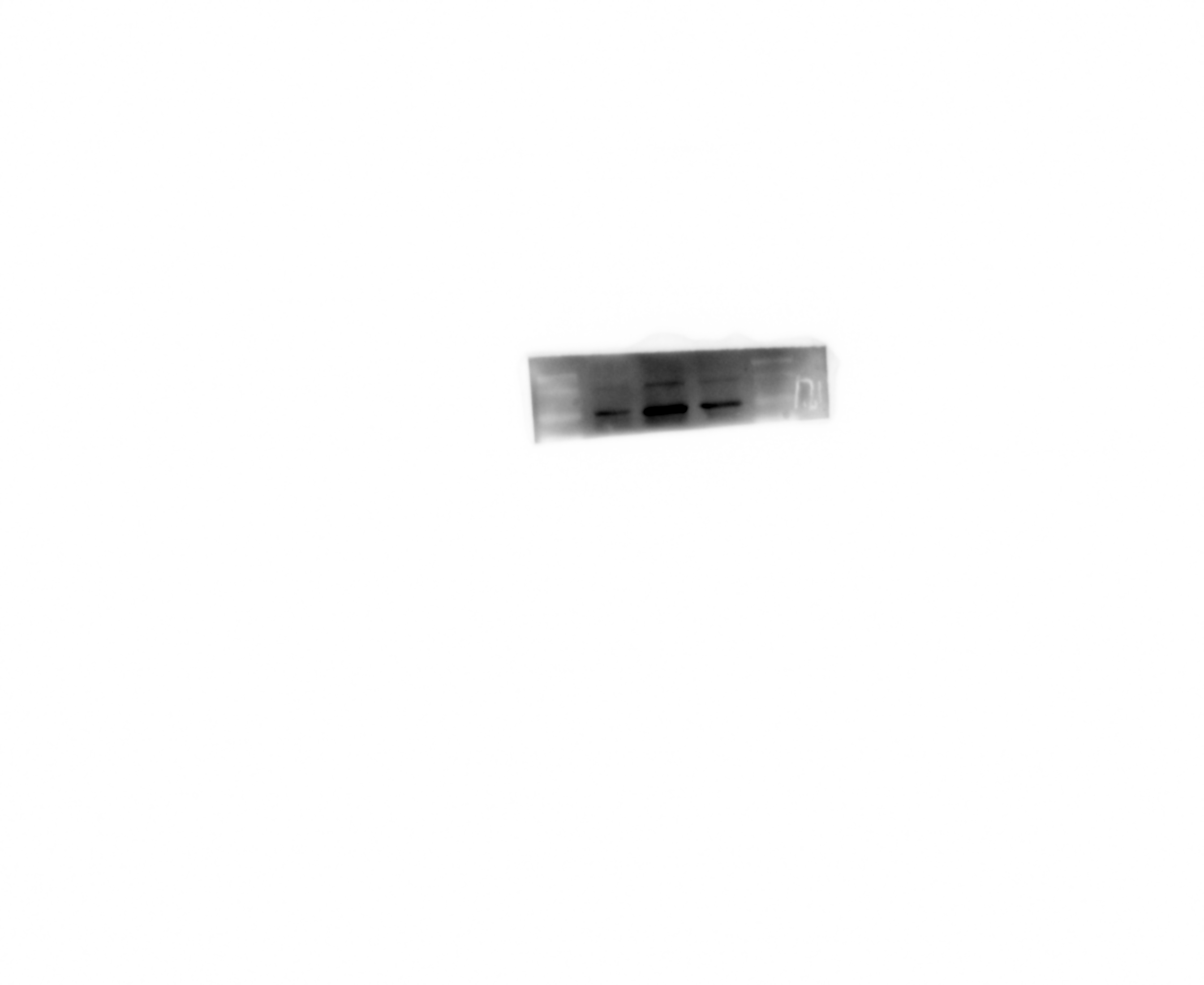

Supplement: Supplemental Information 3 [file peerj-12-17263-s003.zip › Figure 3 WB/002-shine[p-PI3K(1-2-1)0611].jpg]

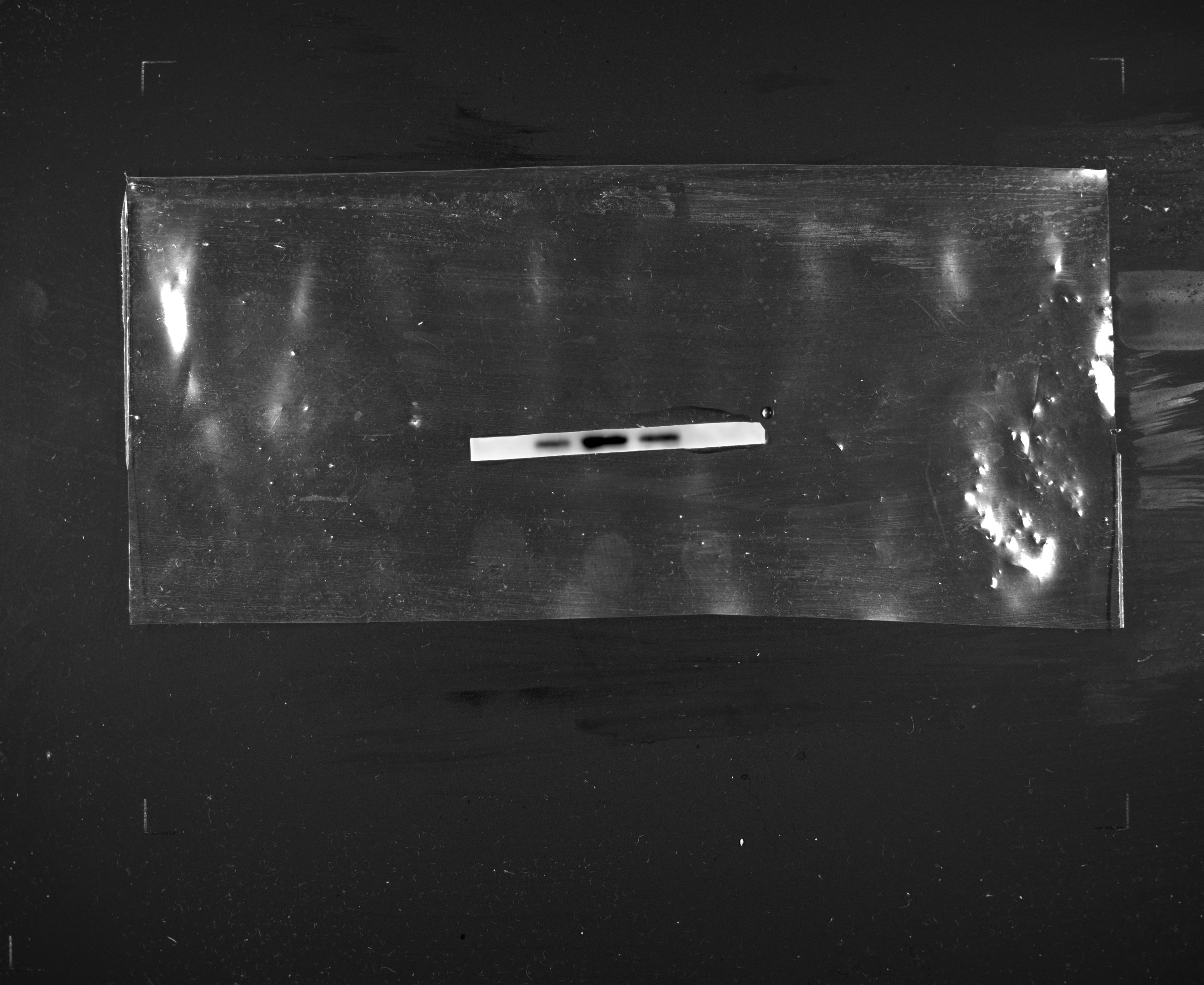

Supplement: Supplemental Information 3 [file peerj-12-17263-s003.zip › Figure 3 WB/003-merger[P-AKT(1-2-2)0611].jpg]

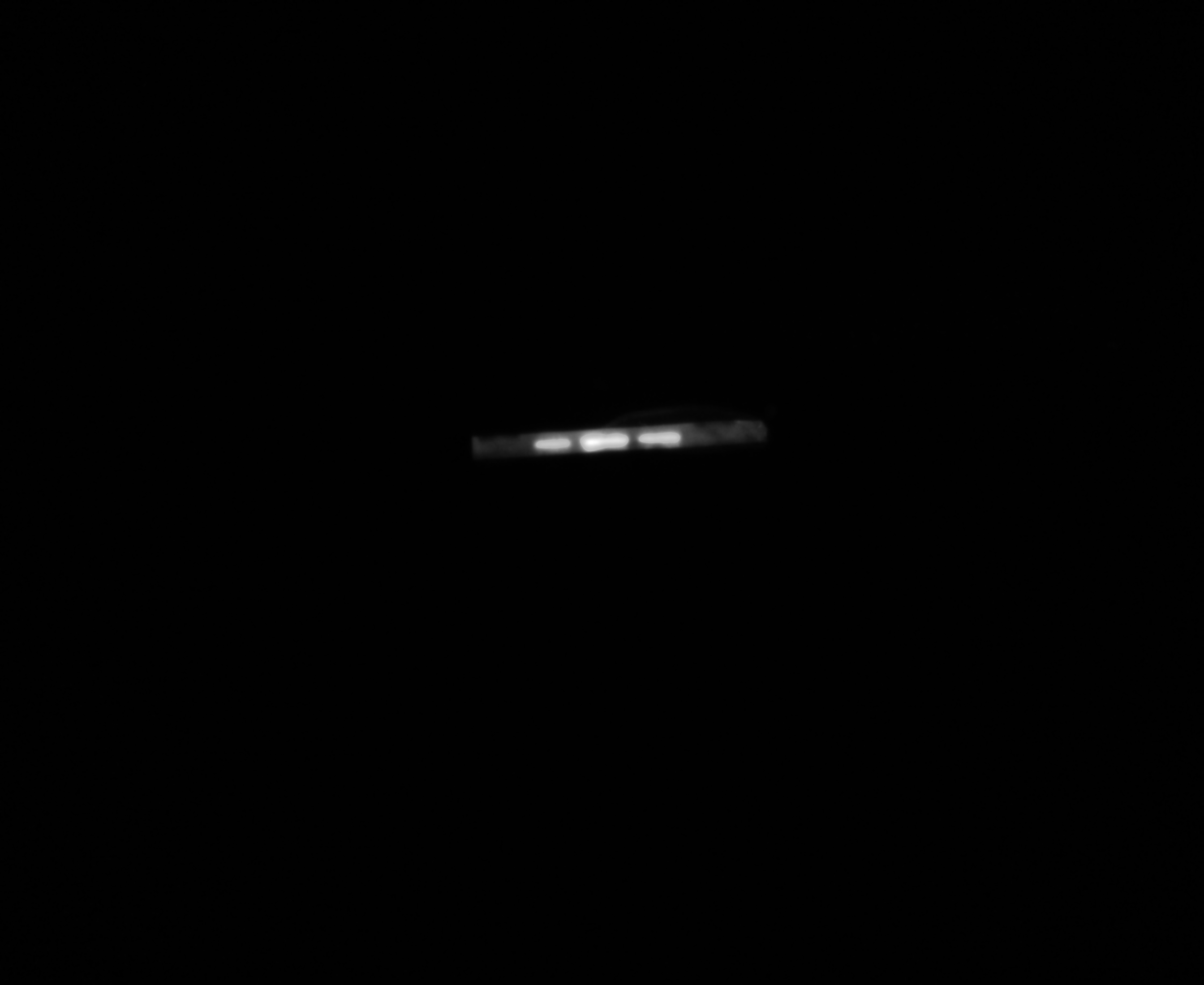

Supplement: Supplemental Information 3 [file peerj-12-17263-s003.zip › Figure 3 WB/003-shine[P-AKT(1-2-2)0611]-raw[366,12182].tif]

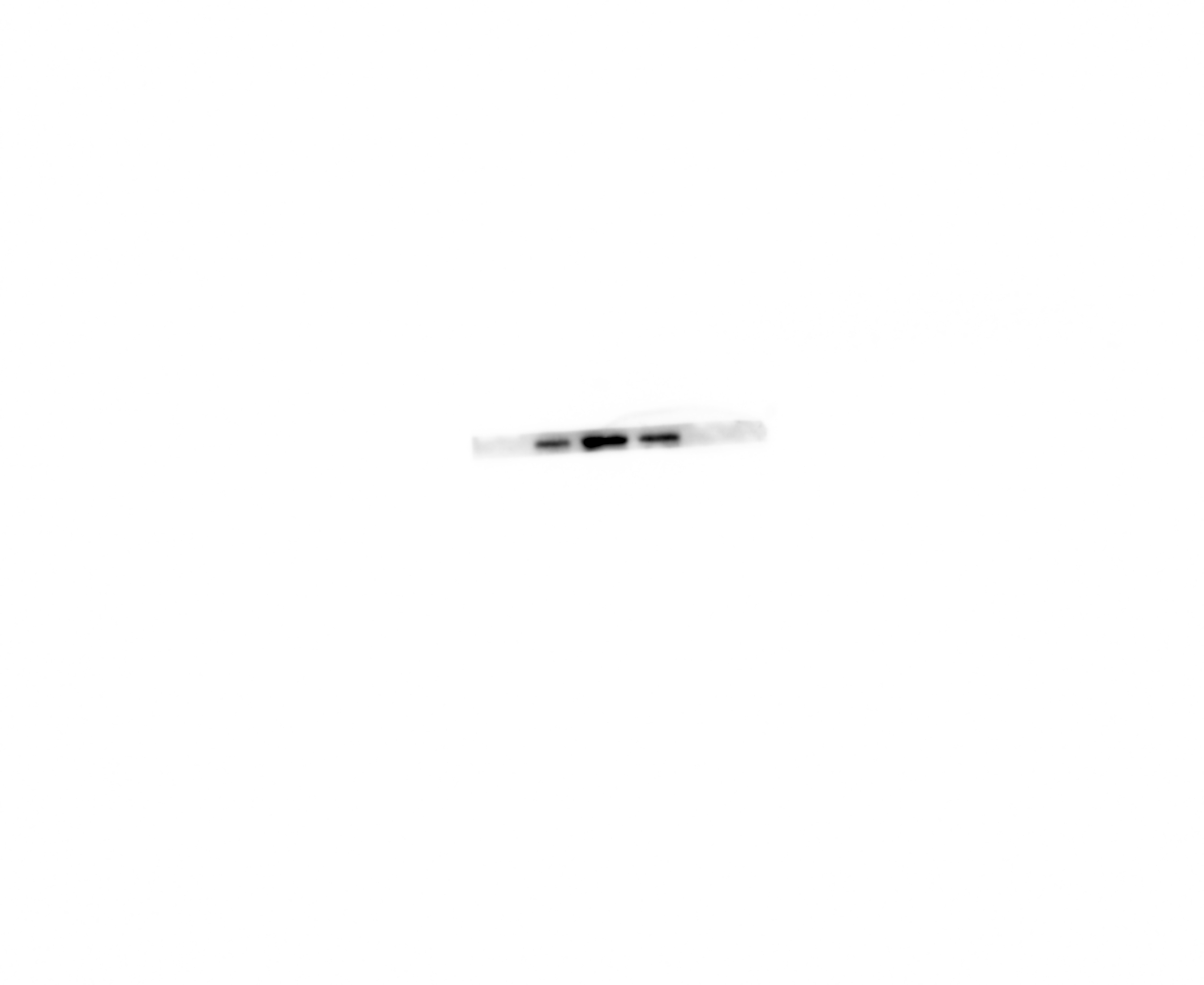

Supplement: Supplemental Information 3 [file peerj-12-17263-s003.zip › Figure 3 WB/003-shine[P-AKT(1-2-2)0611].jpg]

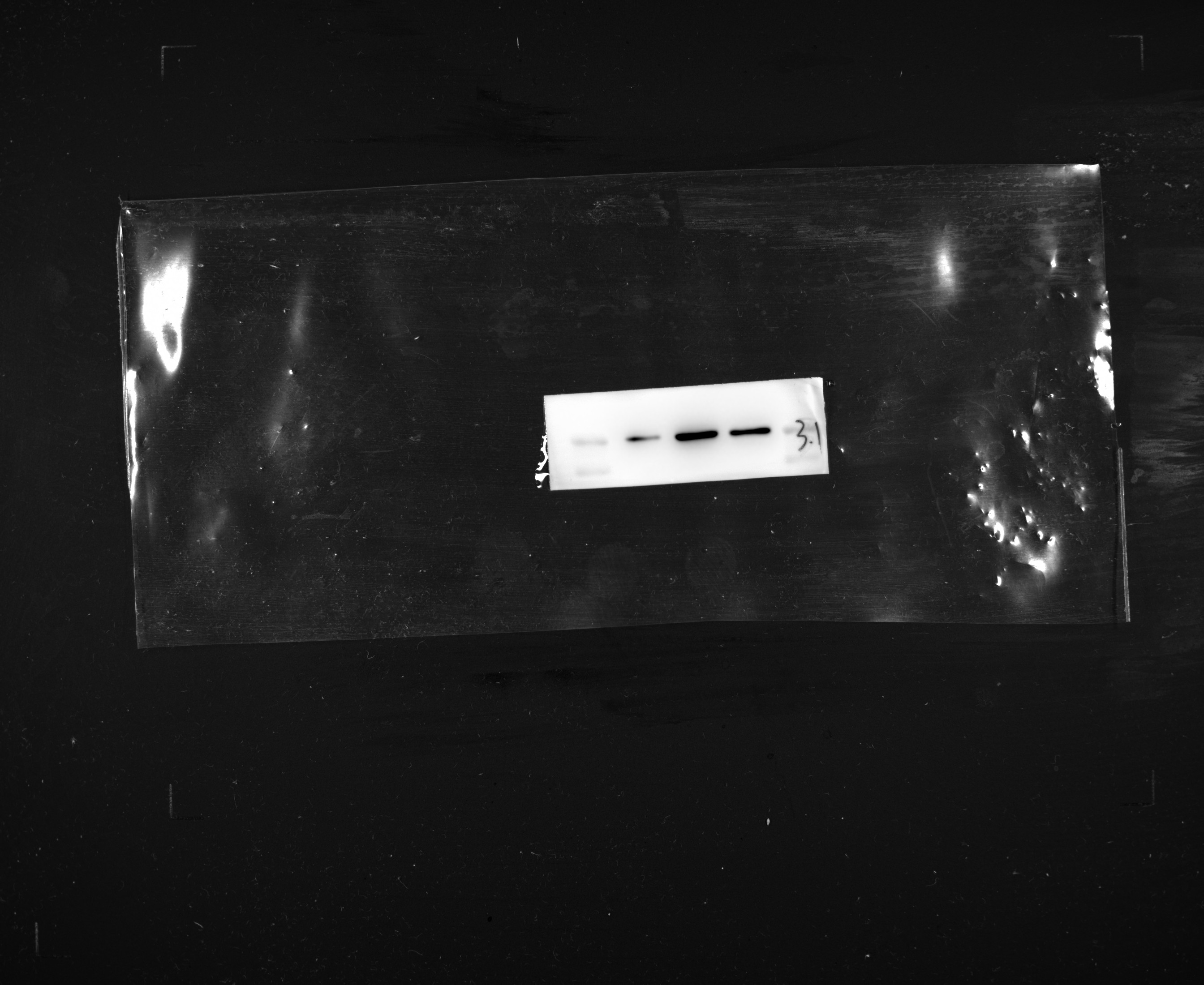

Supplement: Supplemental Information 3 [file peerj-12-17263-s003.zip › Figure 3 WB/004-merger[p-mTOR(3-1)0611].jpg]

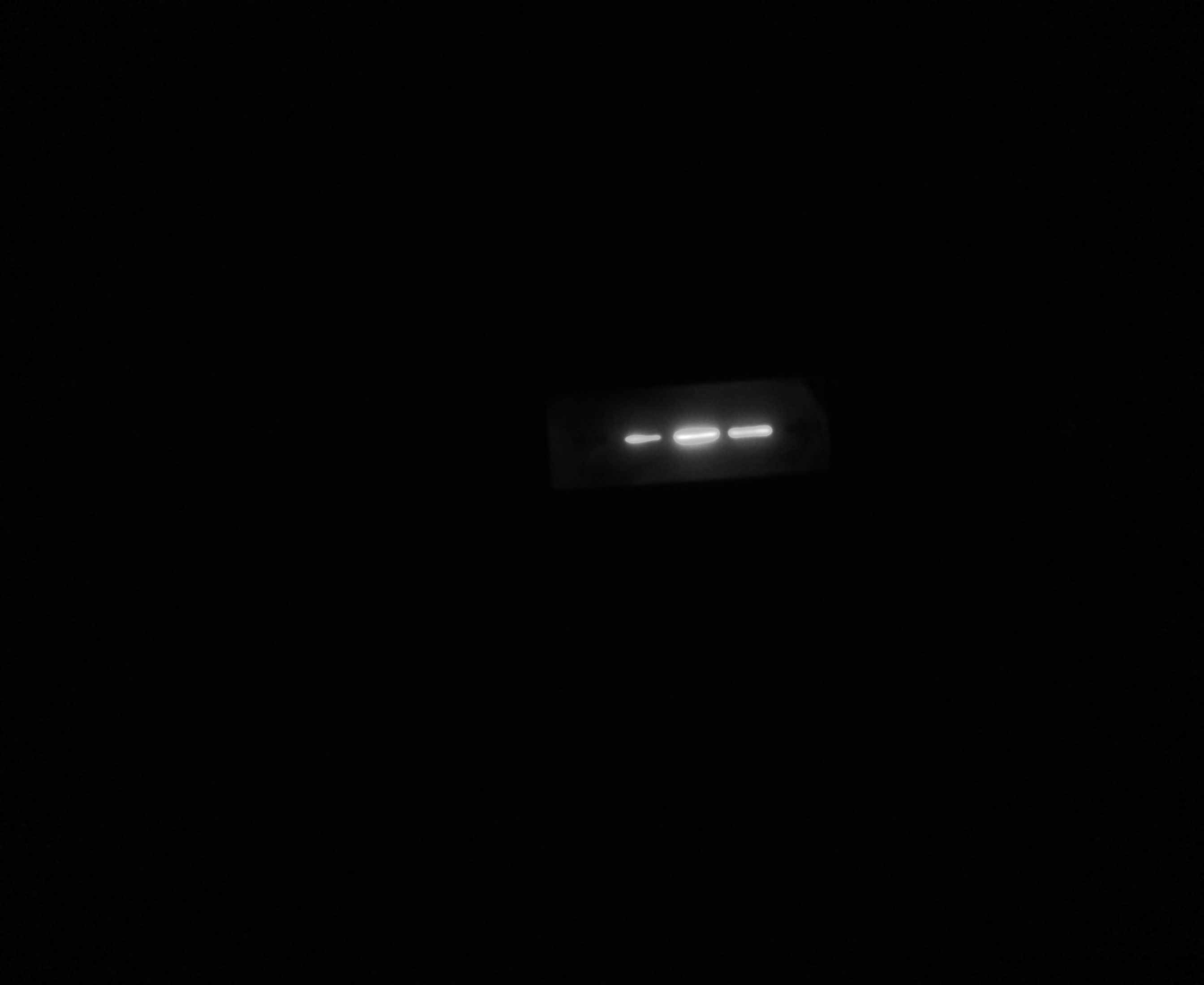

Supplement: Supplemental Information 3 [file peerj-12-17263-s003.zip › Figure 3 WB/004-shine[p-mTOR(3-1)0611]-raw[366,8305].tif]

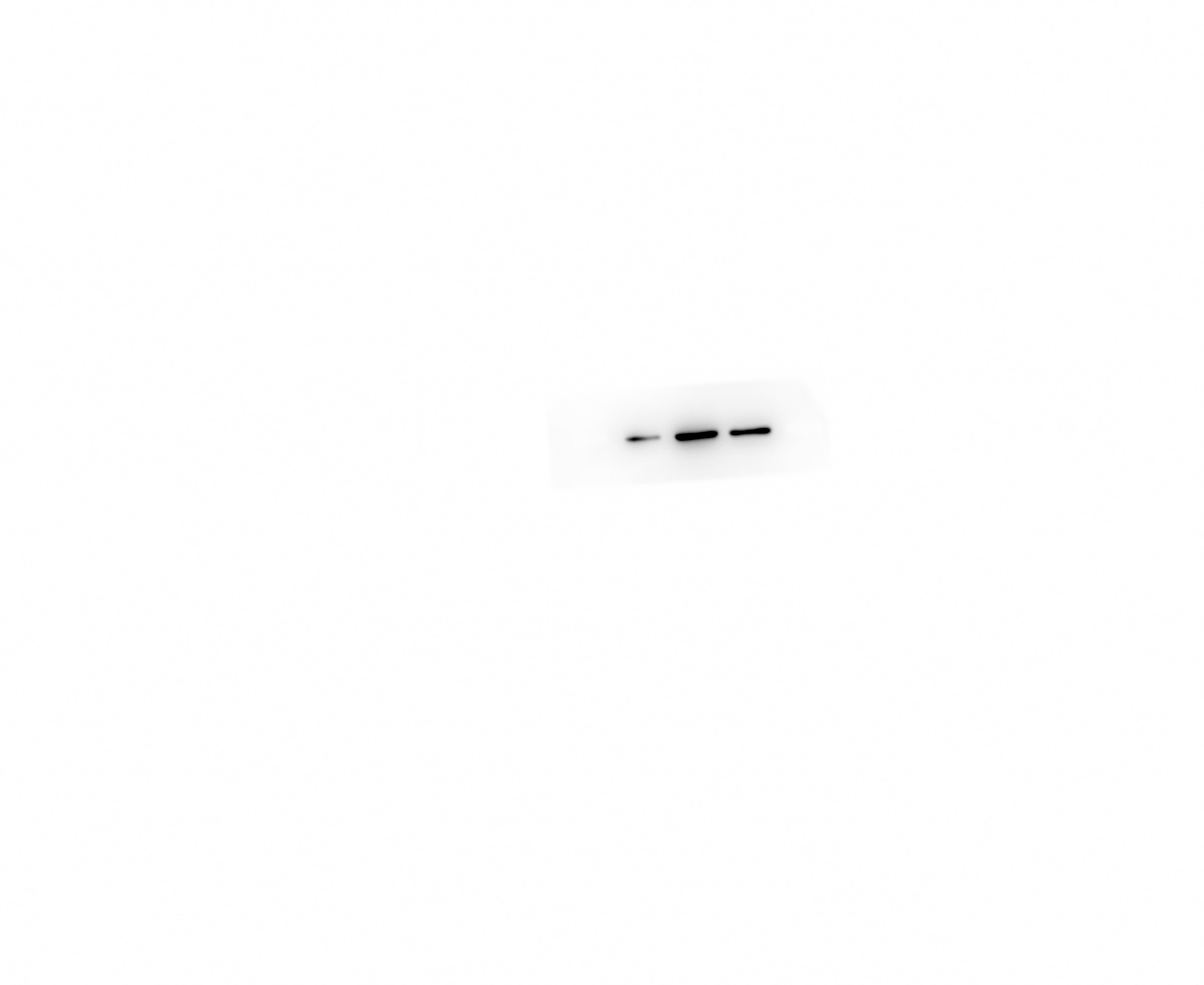

Supplement: Supplemental Information 3 [file peerj-12-17263-s003.zip › Figure 3 WB/004-shine[p-mTOR(3-1)0611].jpg]

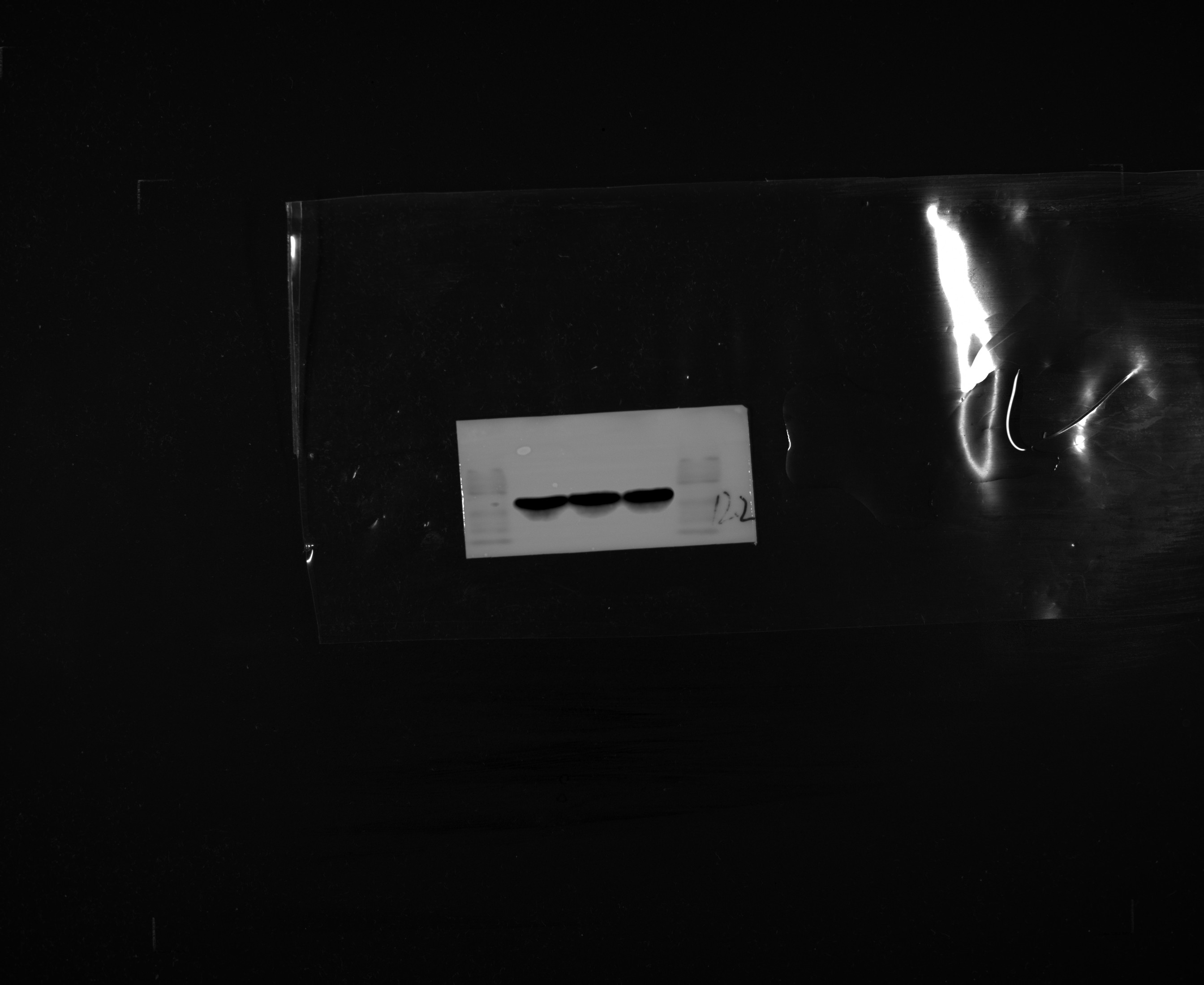

Supplement: Supplemental Information 3 [file peerj-12-17263-s003.zip › Figure 3 WB/005-merger[GAPDH(12-2)1219].jpg]

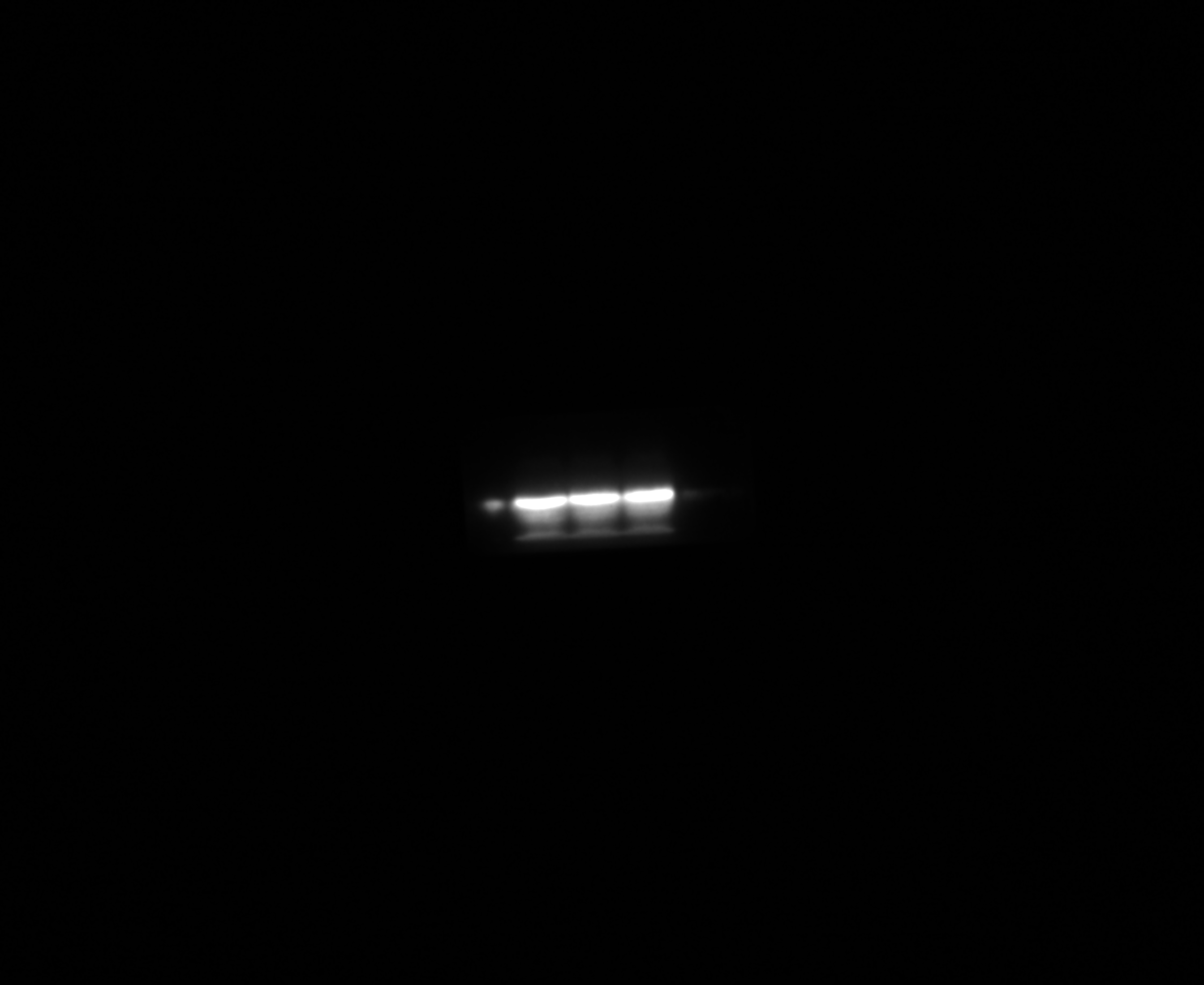

Supplement: Supplemental Information 3 [file peerj-12-17263-s003.zip › Figure 3 WB/005-shine[GAPDH(12-2)1219]-raw[368,10938].tif]

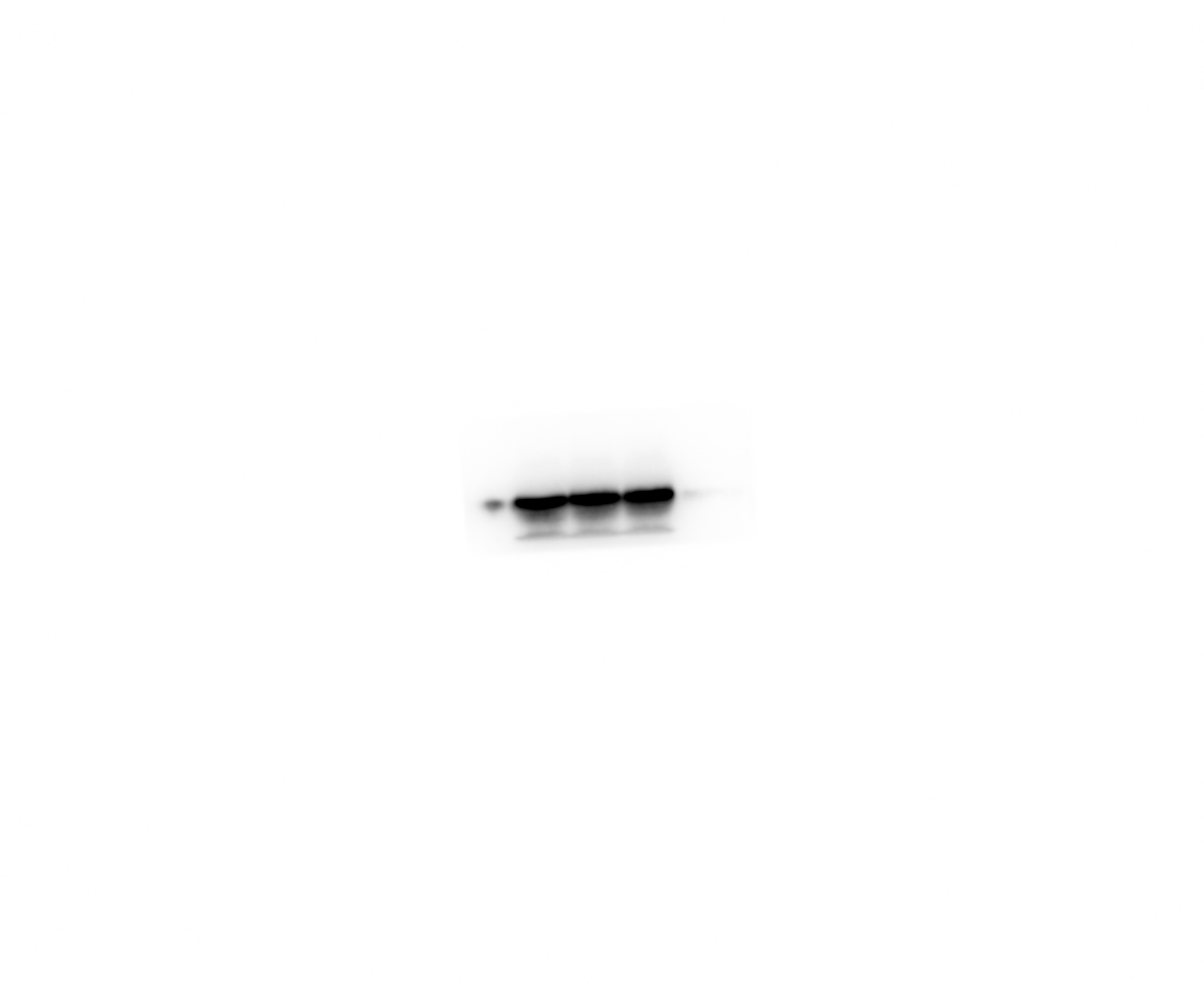

Supplement: Supplemental Information 3 [file peerj-12-17263-s003.zip › Figure 3 WB/005-shine[GAPDH(12-2)1219].jpg]

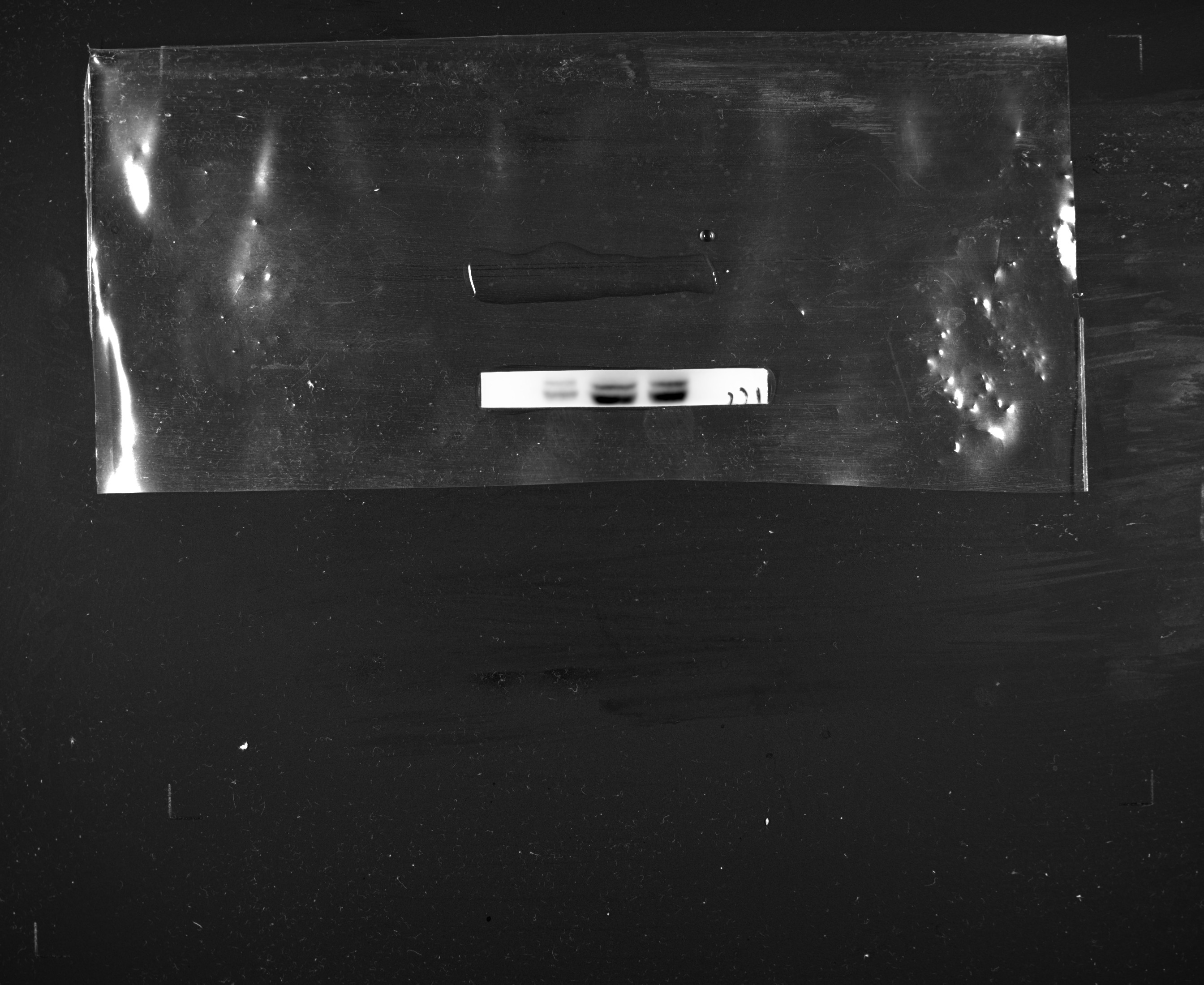

Supplement: Supplemental Information 4 [file peerj-12-17263-s004.zip › Figure 4 WB/001-merger[LC3I-II(8-2)0611].jpg]

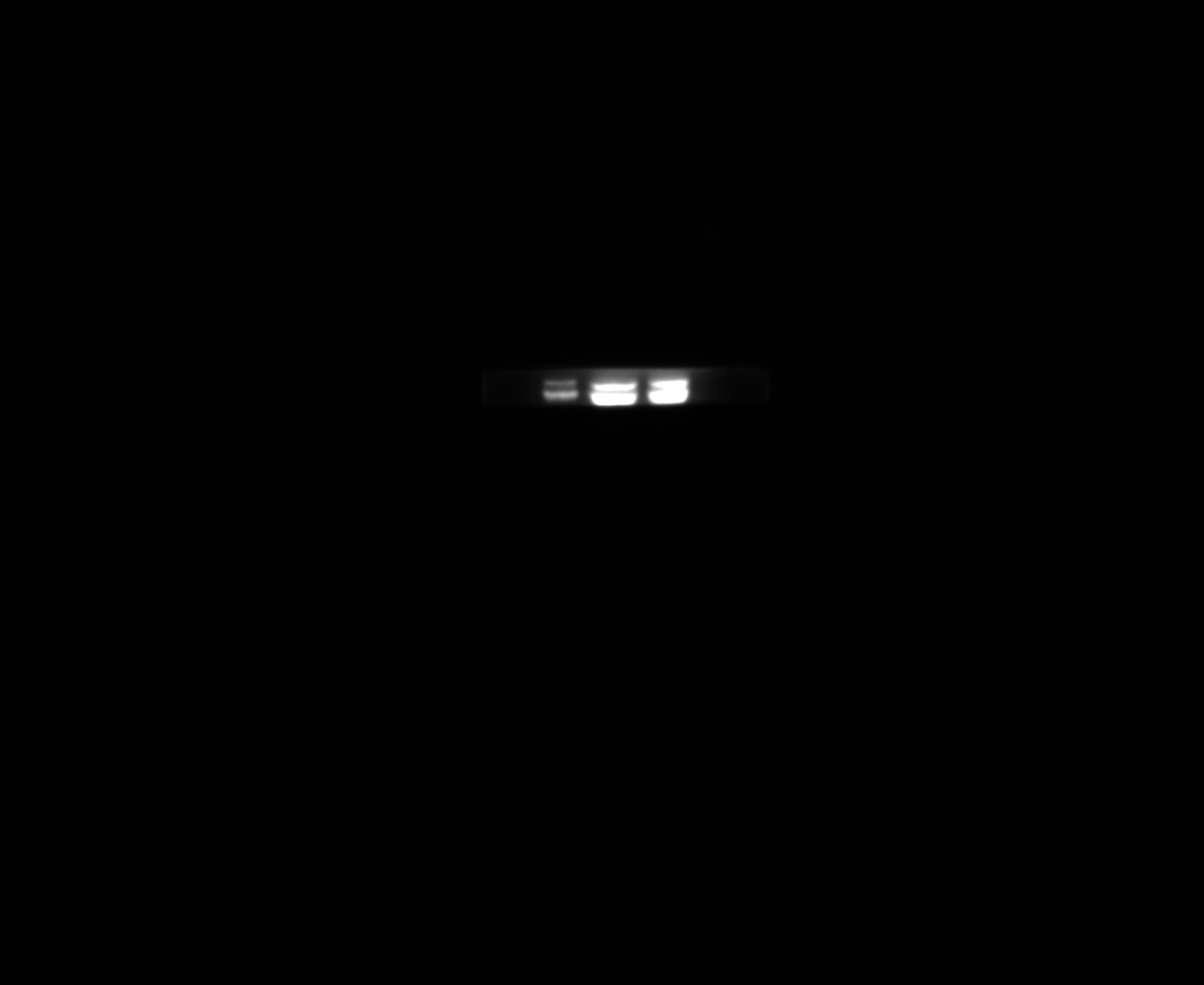

Supplement: Supplemental Information 4 [file peerj-12-17263-s004.zip › Figure 4 WB/001-shine[LC3I-II(8-2)0611]-raw[366,16313].tif]

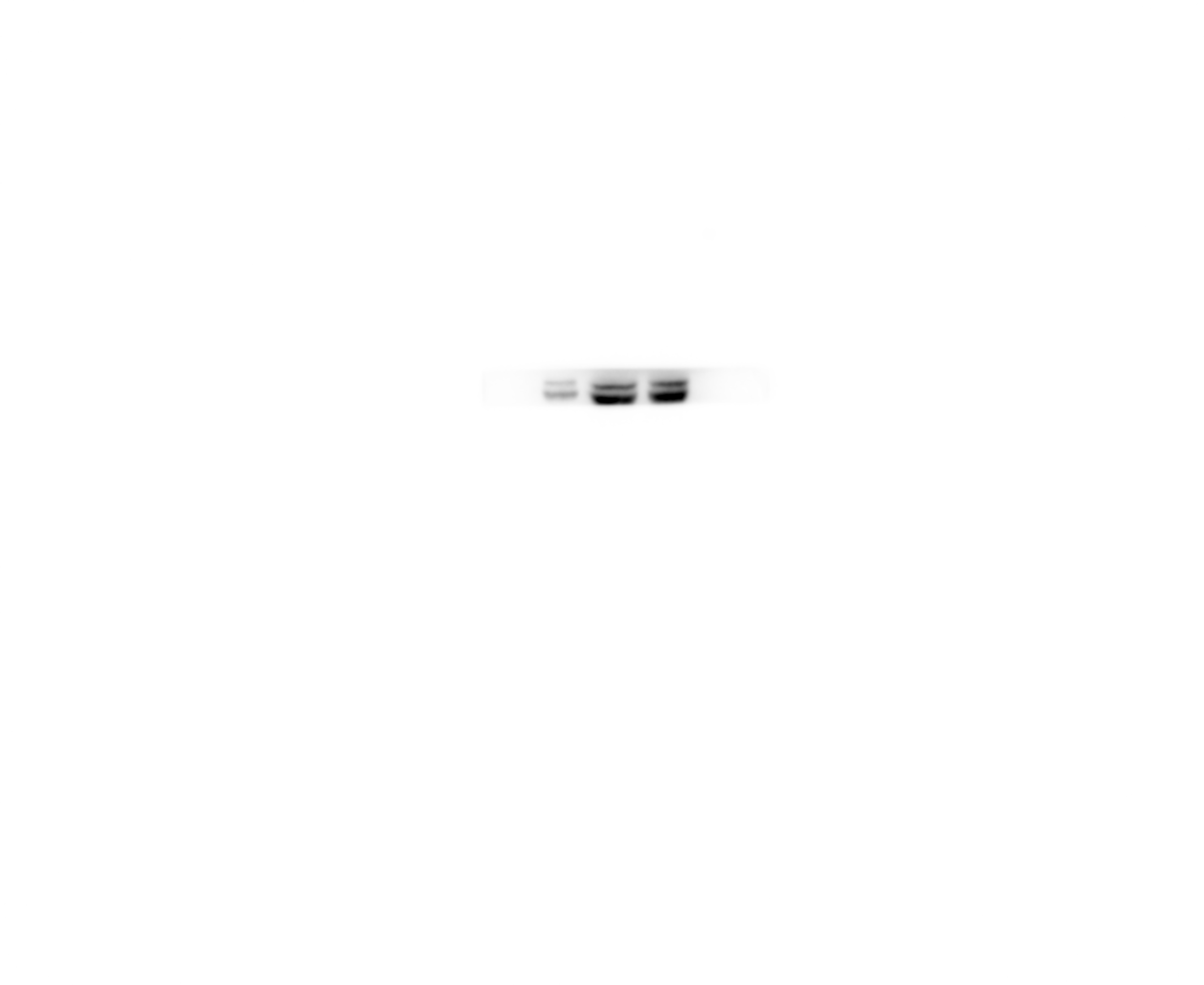

Supplement: Supplemental Information 4 [file peerj-12-17263-s004.zip › Figure 4 WB/001-shine[LC3I-II(8-2)0611].jpg]

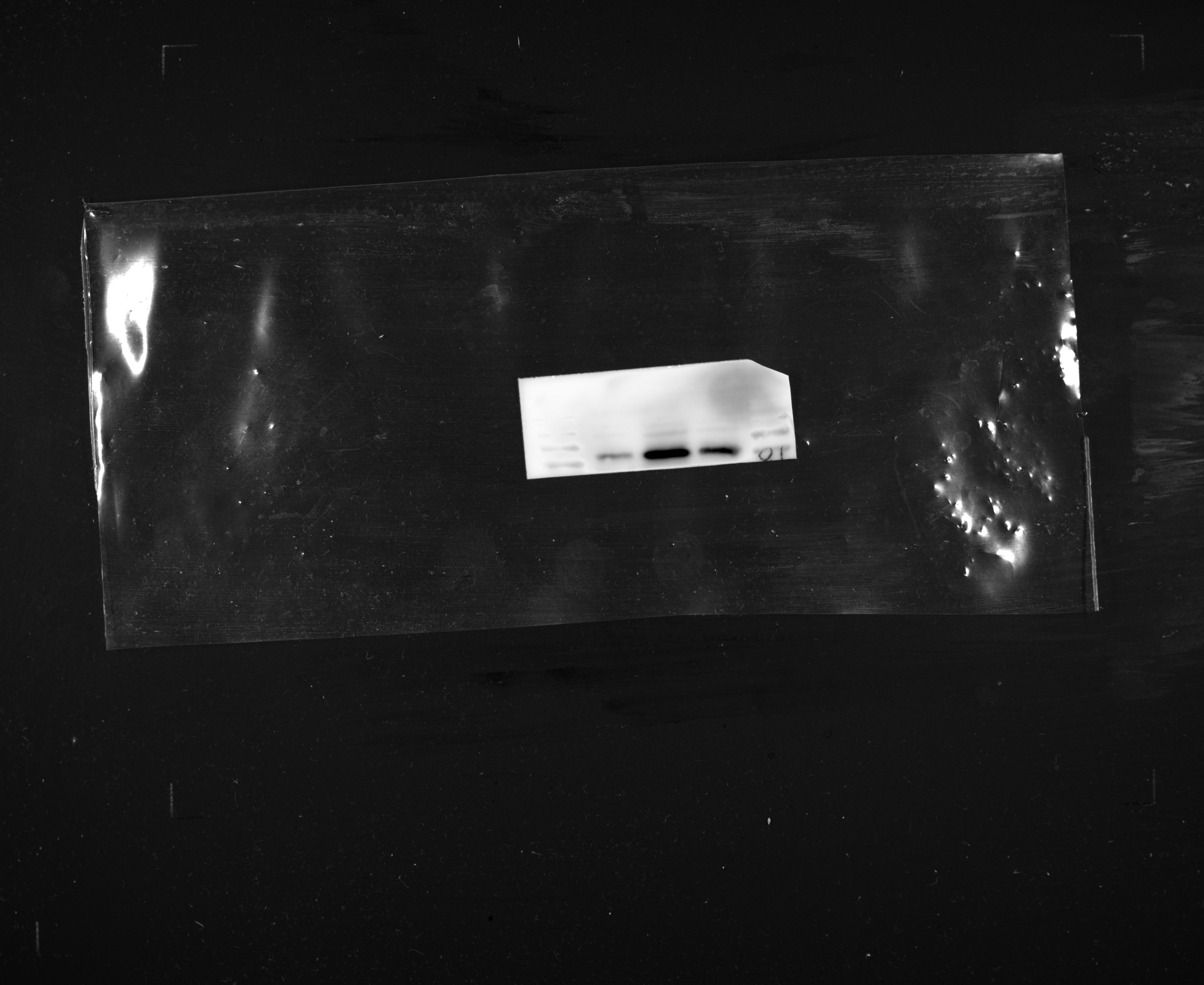

Supplement: Supplemental Information 4 [file peerj-12-17263-s004.zip › Figure 4 WB/002-merger[Beclin(8-1)0611].jpg]

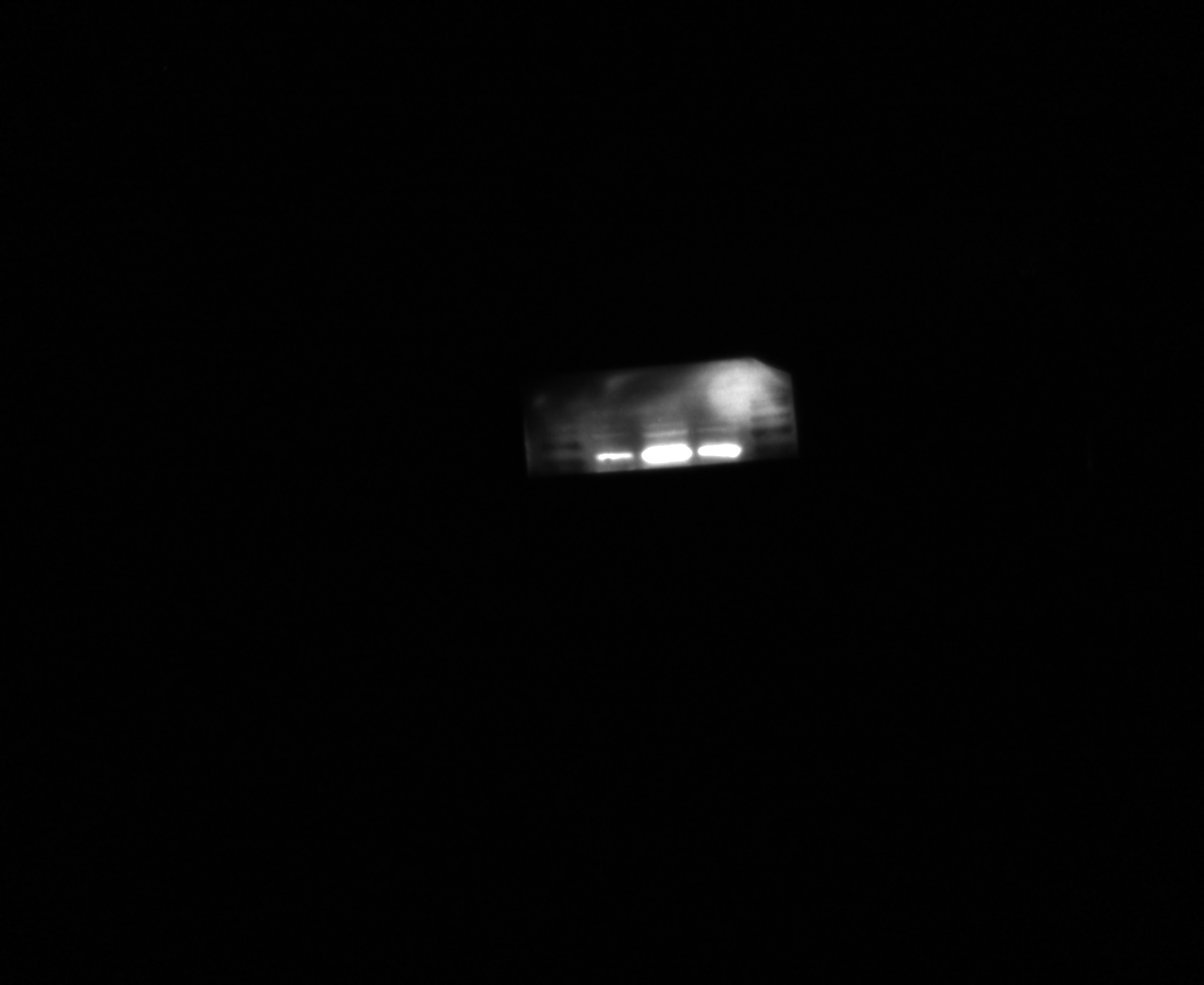

Supplement: Supplemental Information 4 [file peerj-12-17263-s004.zip › Figure 4 WB/002-shine[Beclin(8-1)0611]-raw[366,13542].tif]

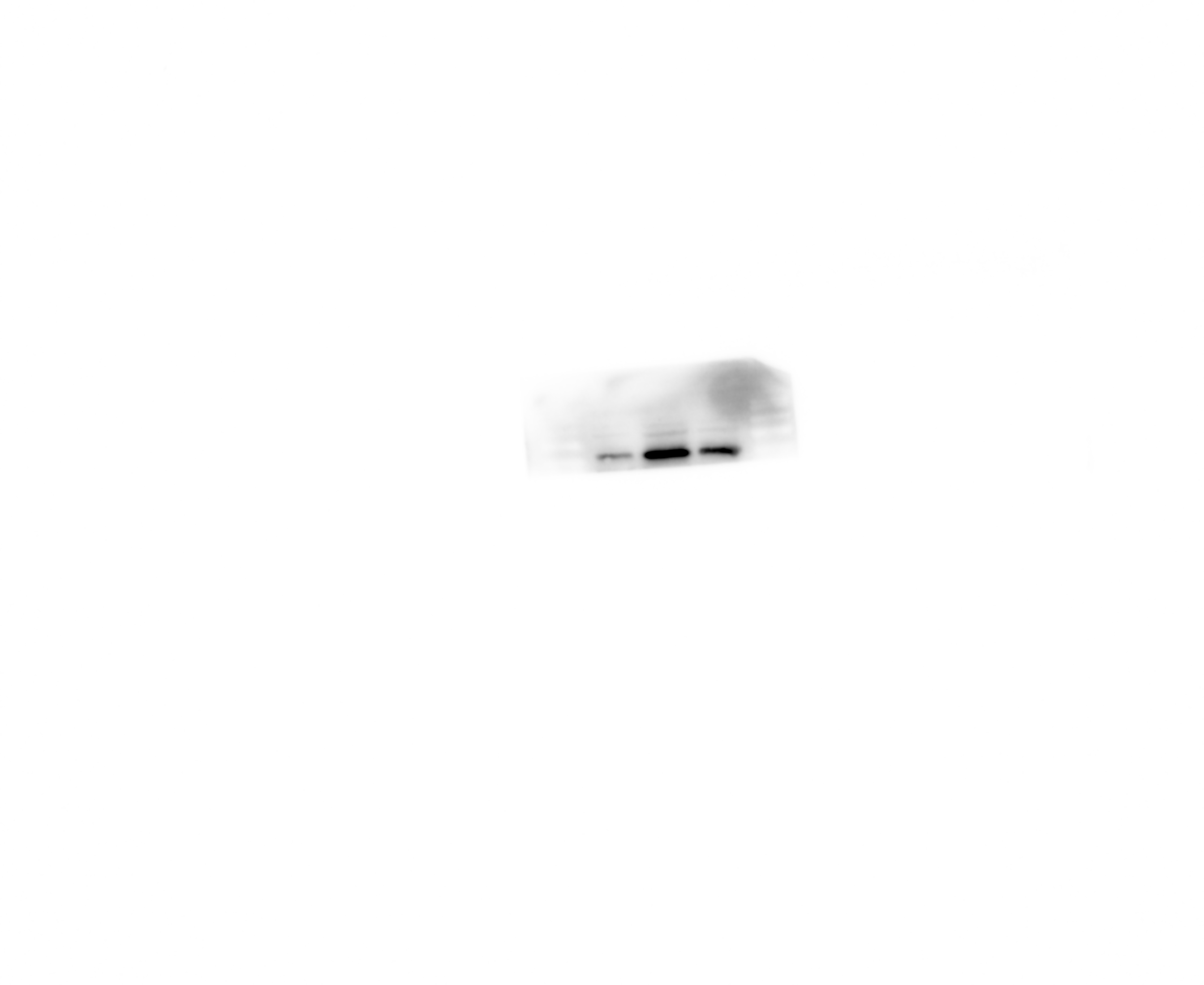

Supplement: Supplemental Information 4 [file peerj-12-17263-s004.zip › Figure 4 WB/002-shine[Beclin(8-1)0611].jpg]

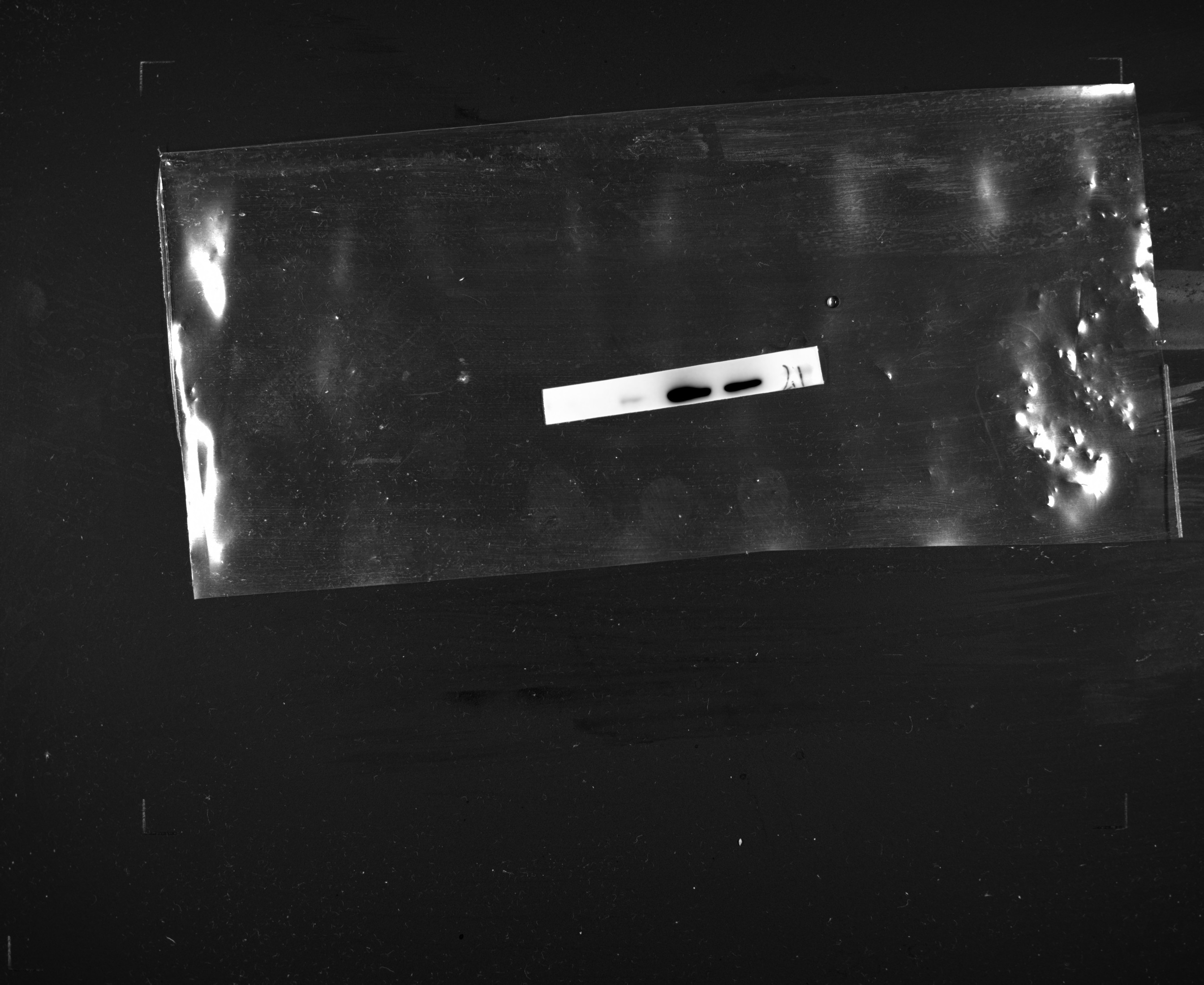

Supplement: Supplemental Information 4 [file peerj-12-17263-s004.zip › Figure 4 WB/003-merger[Atg5(2-1)0611].jpg]

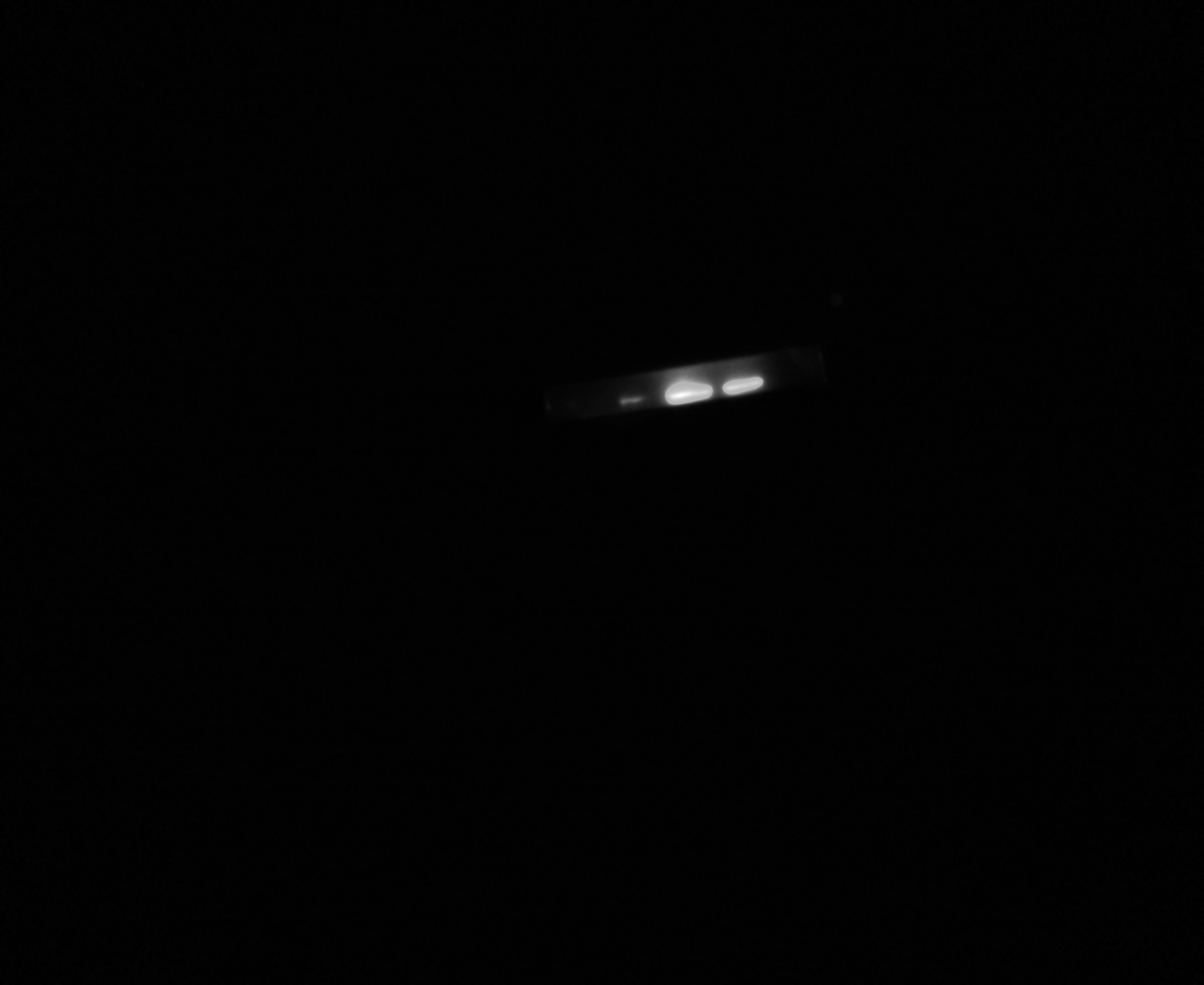

Supplement: Supplemental Information 4 [file peerj-12-17263-s004.zip › Figure 4 WB/003-shine[Atg5(2-1)0611]-raw[369,7363].tif]

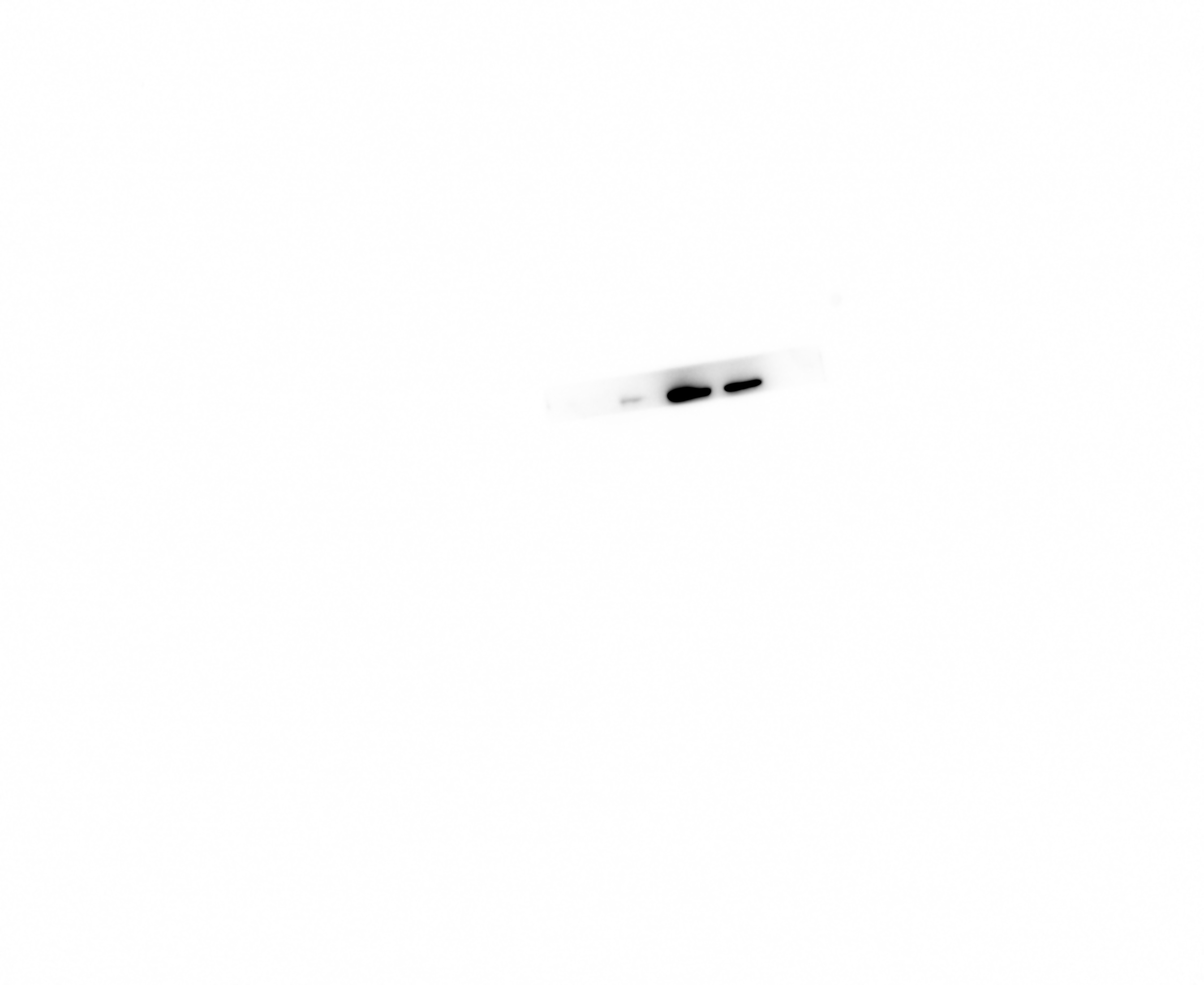

Supplement: Supplemental Information 4 [file peerj-12-17263-s004.zip › Figure 4 WB/003-shine[Atg5(2-1)0611].jpg]

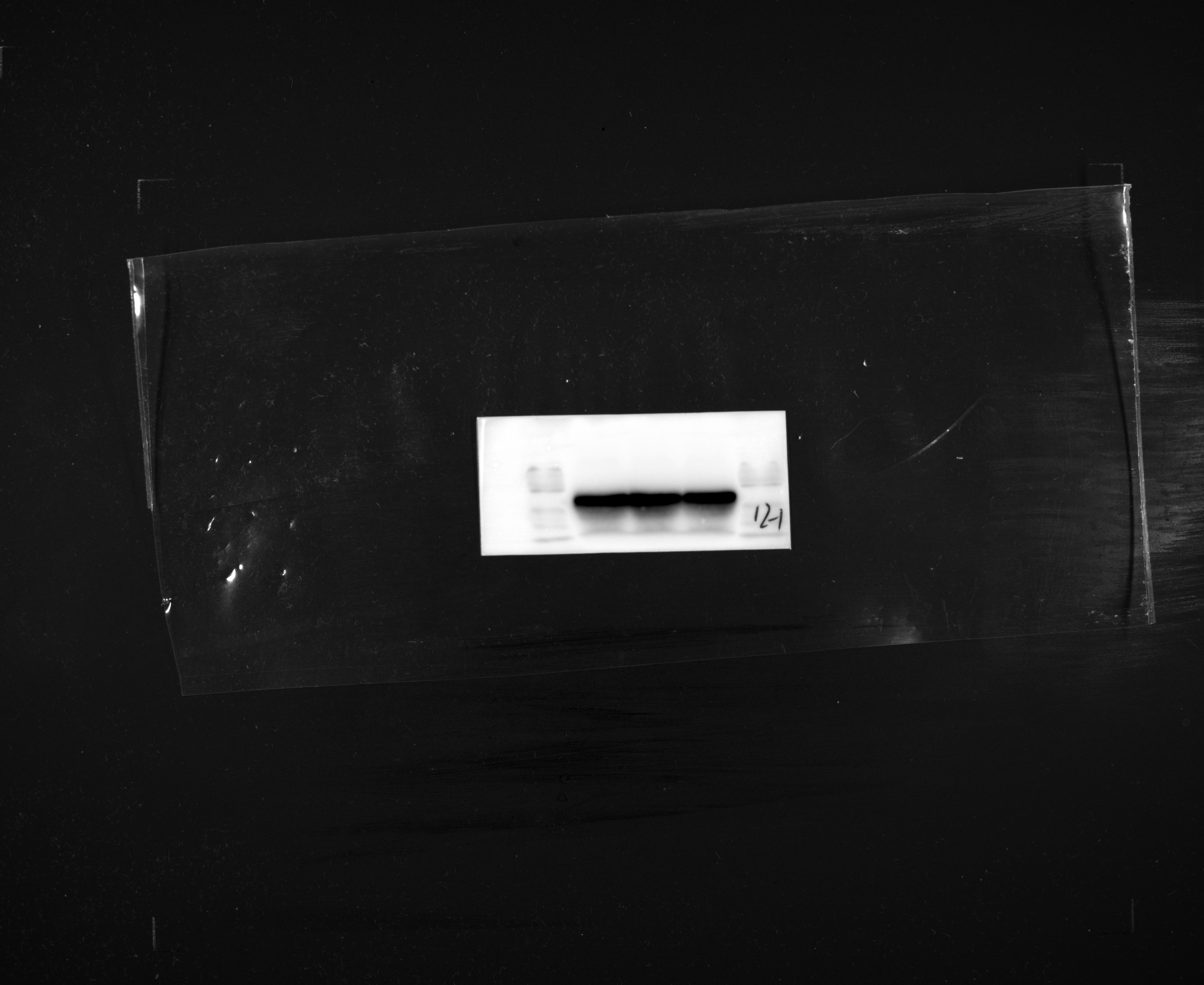

Supplement: Supplemental Information 4 [file peerj-12-17263-s004.zip › Figure 4 WB/004-merger[GAPDH(12-1)1219].jpg]

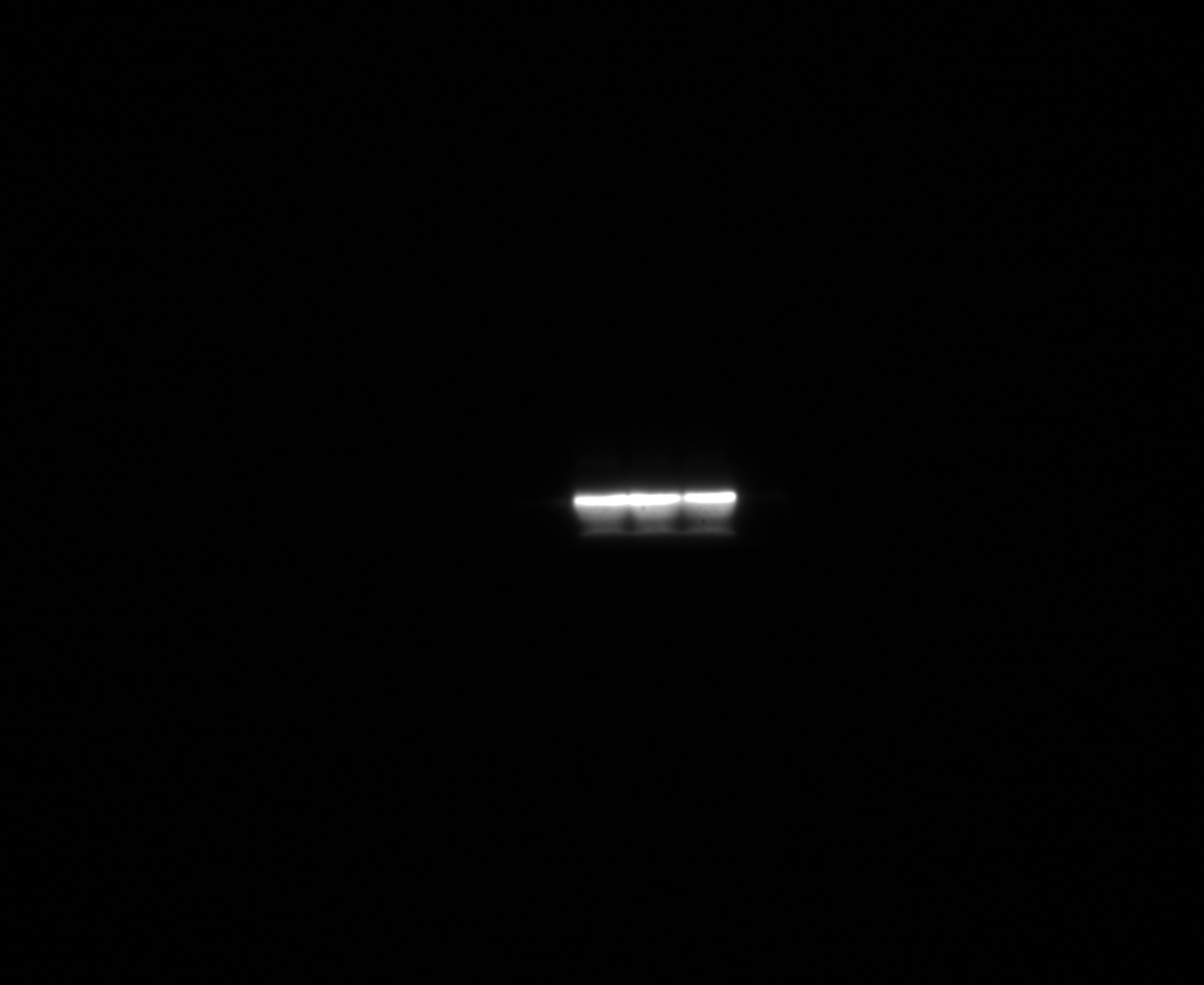

Supplement: Supplemental Information 4 [file peerj-12-17263-s004.zip › Figure 4 WB/004-shine[GAPDH(12-1)1219]-raw[368,10079].tif]

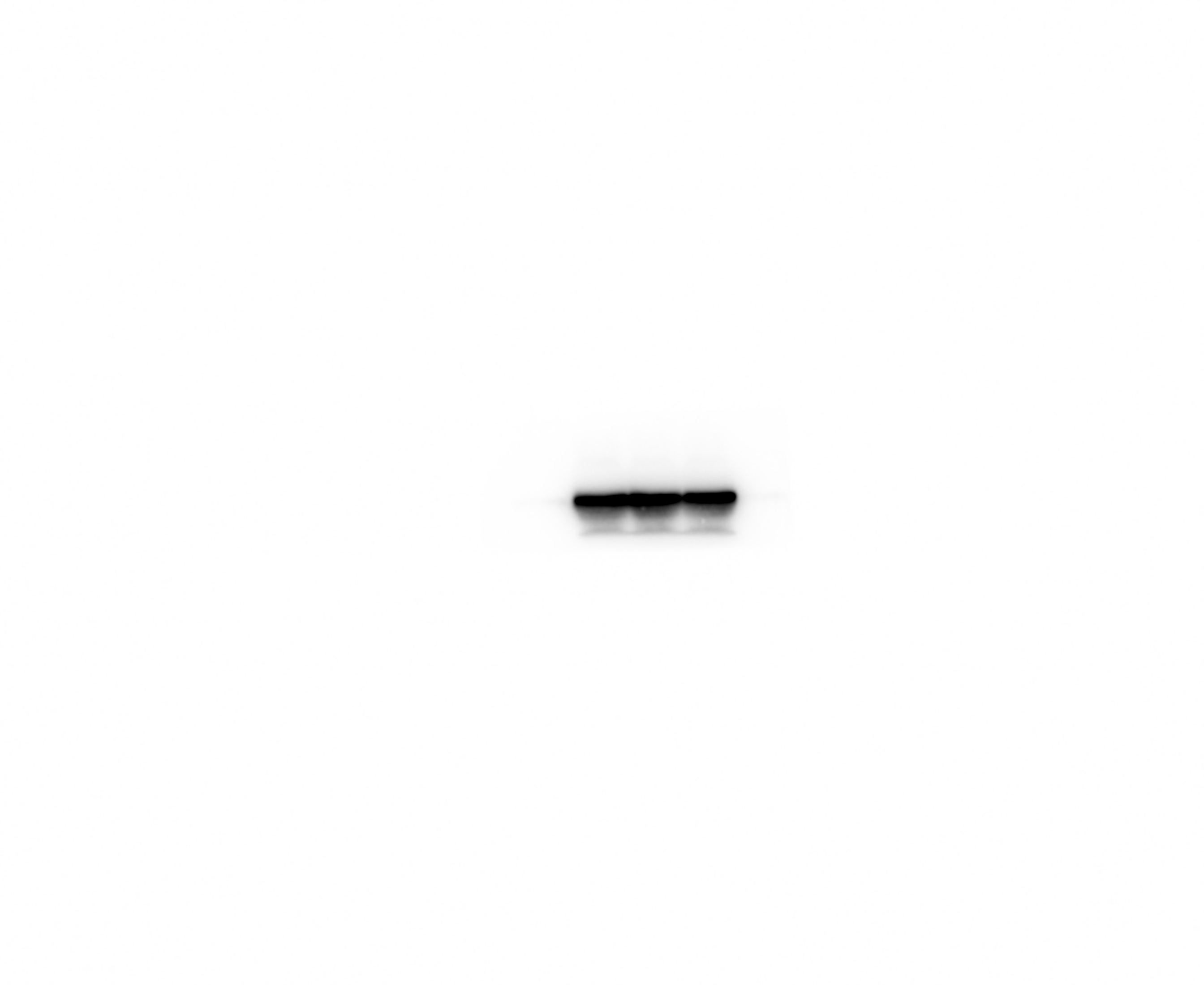

Supplement: Supplemental Information 4 [file peerj-12-17263-s004.zip › Figure 4 WB/004-shine[GAPDH(12-1)1219].jpg]

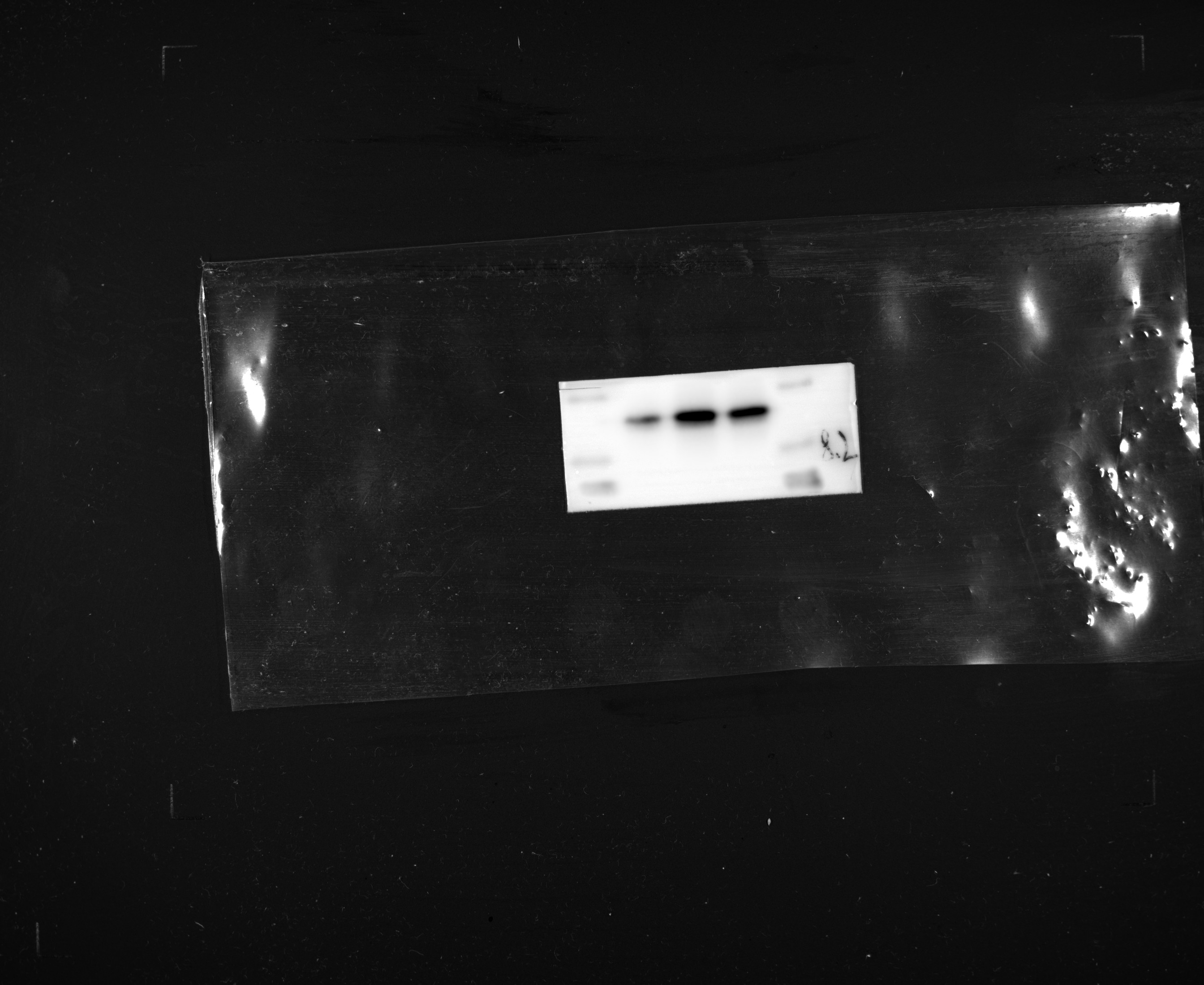

Supplement: Supplemental Information 5 [file peerj-12-17263-s005.zip › Figure 5 wb/001-merger[BAX(8-2)0611].jpg]

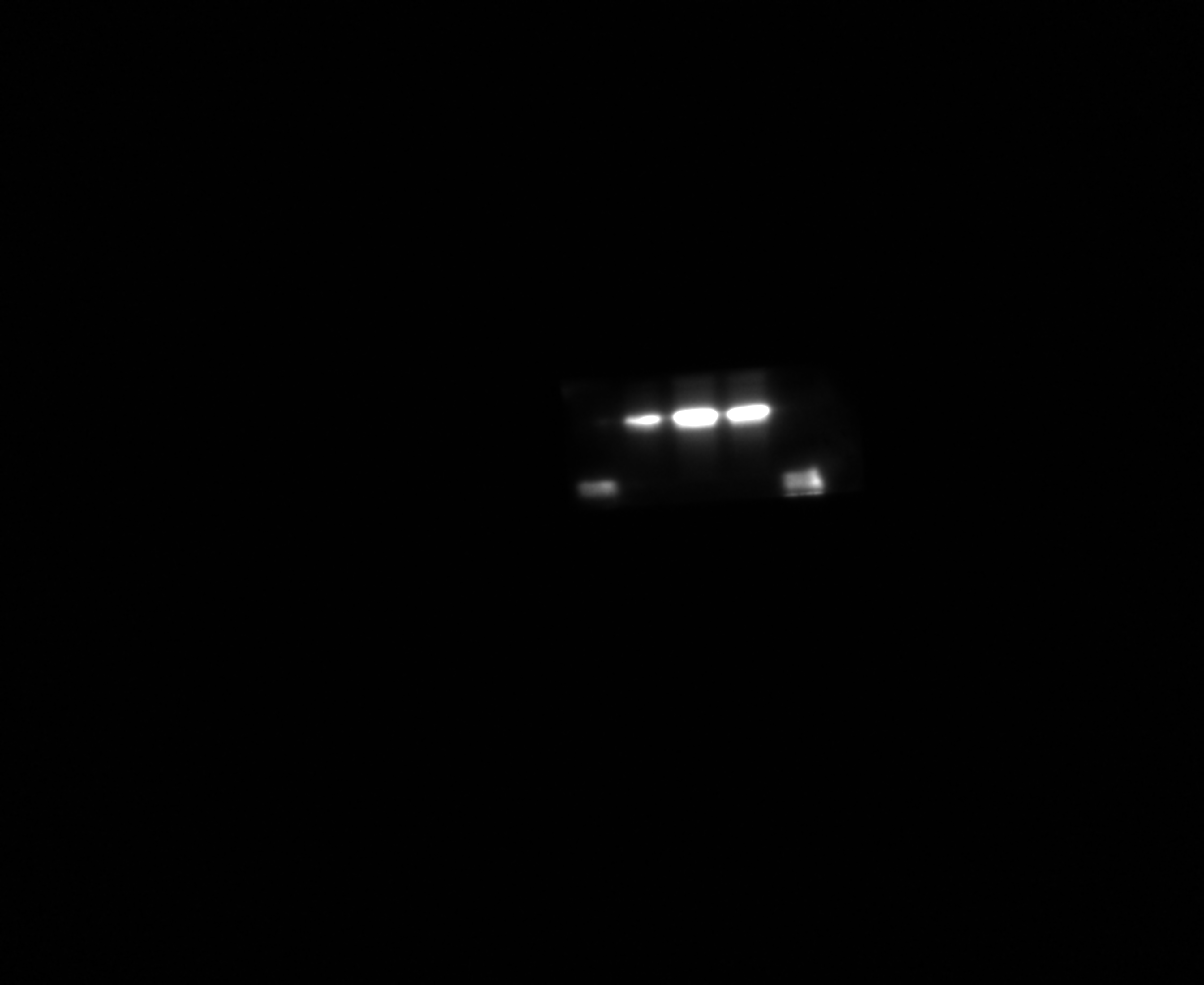

Supplement: Supplemental Information 5 [file peerj-12-17263-s005.zip › Figure 5 wb/001-shine[BAX(8-2)0611]-raw[368,14057].tif]

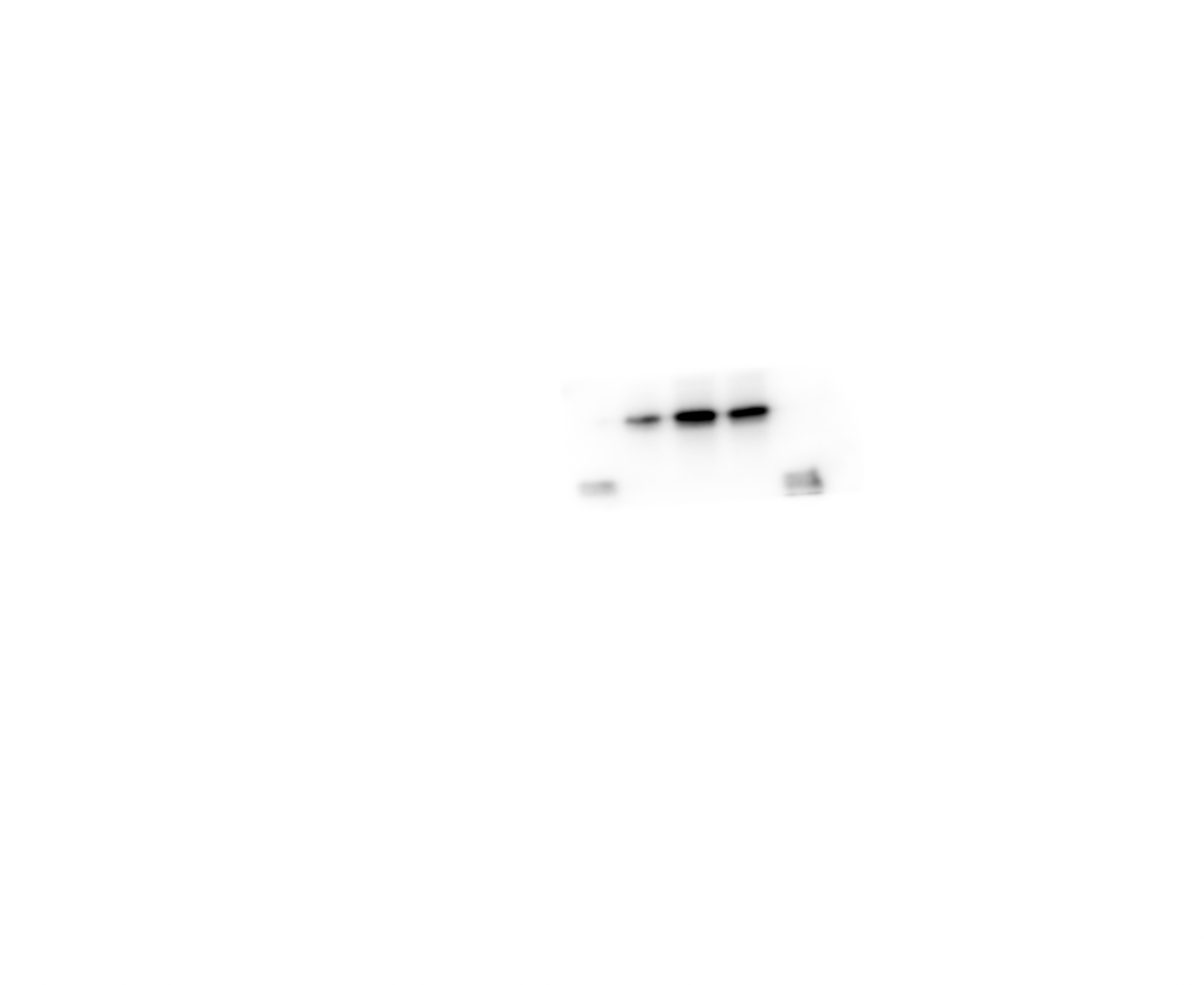

Supplement: Supplemental Information 5 [file peerj-12-17263-s005.zip › Figure 5 wb/001-shine[BAX(8-2)0611].jpg]

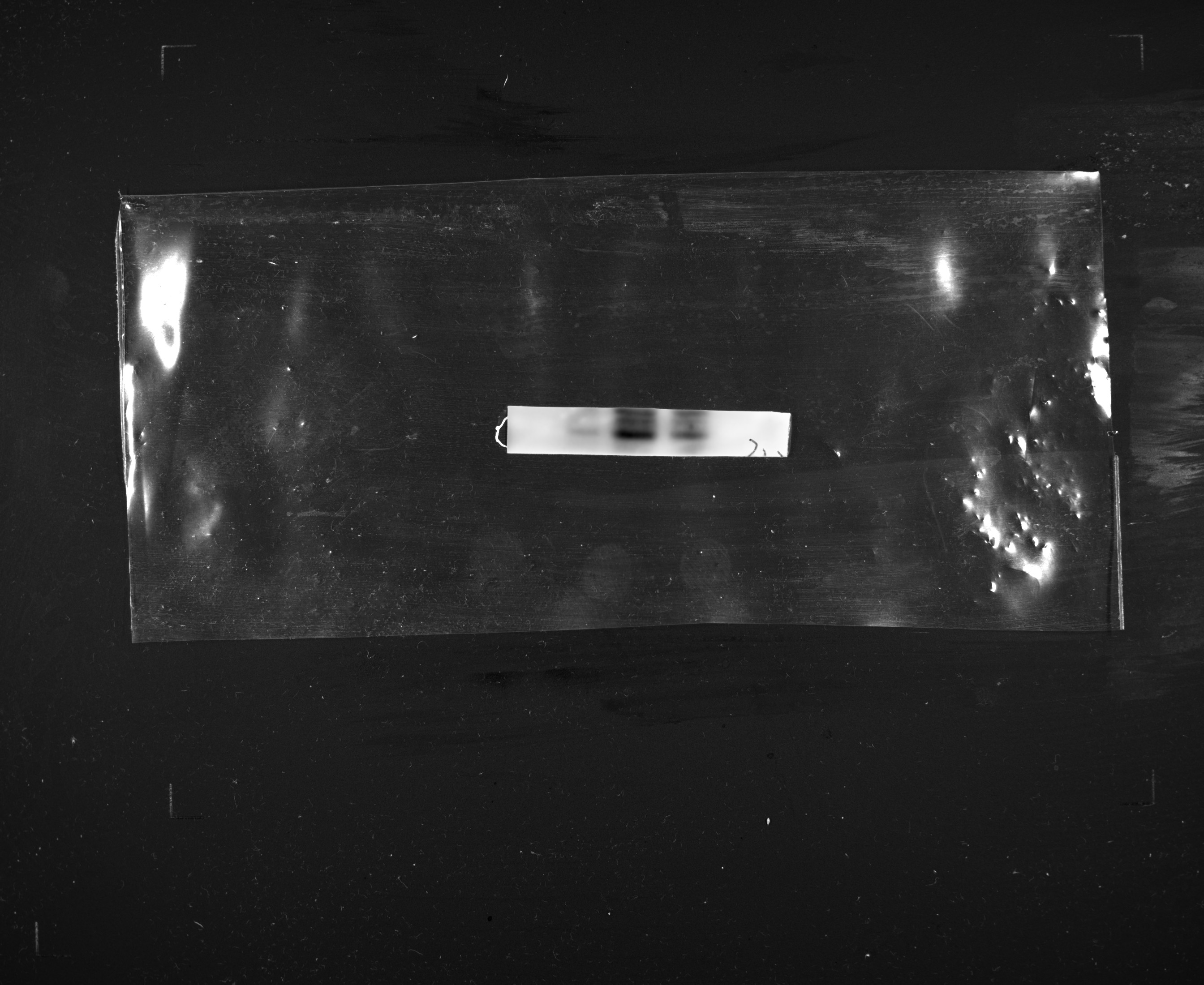

Supplement: Supplemental Information 5 [file peerj-12-17263-s005.zip › Figure 5 wb/002-merger[caspase3(2-2-2)0611].jpg]

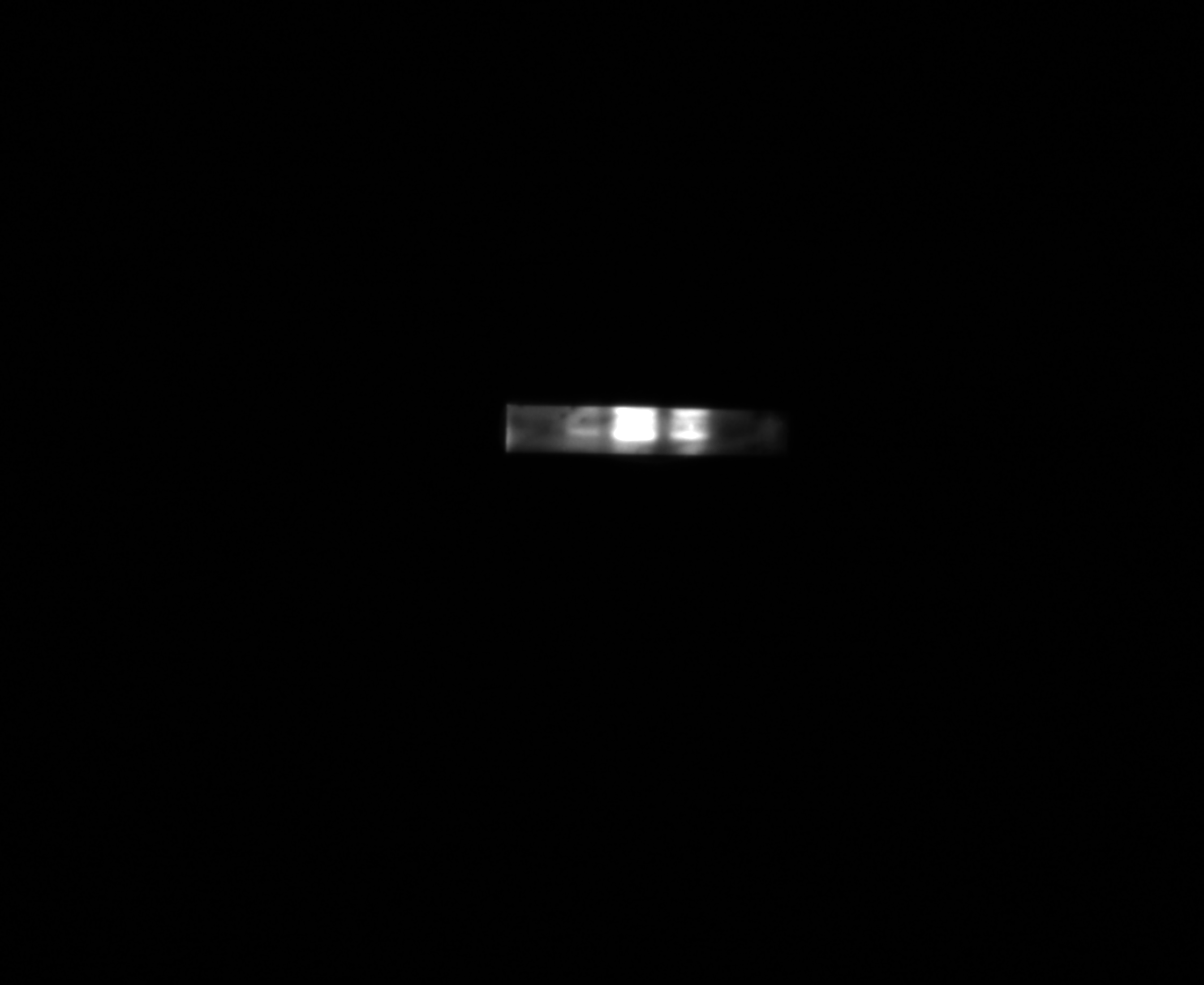

Supplement: Supplemental Information 5 [file peerj-12-17263-s005.zip › Figure 5 wb/002-shine[caspase3(2-2-2)0611]-raw[369,18763].tif]

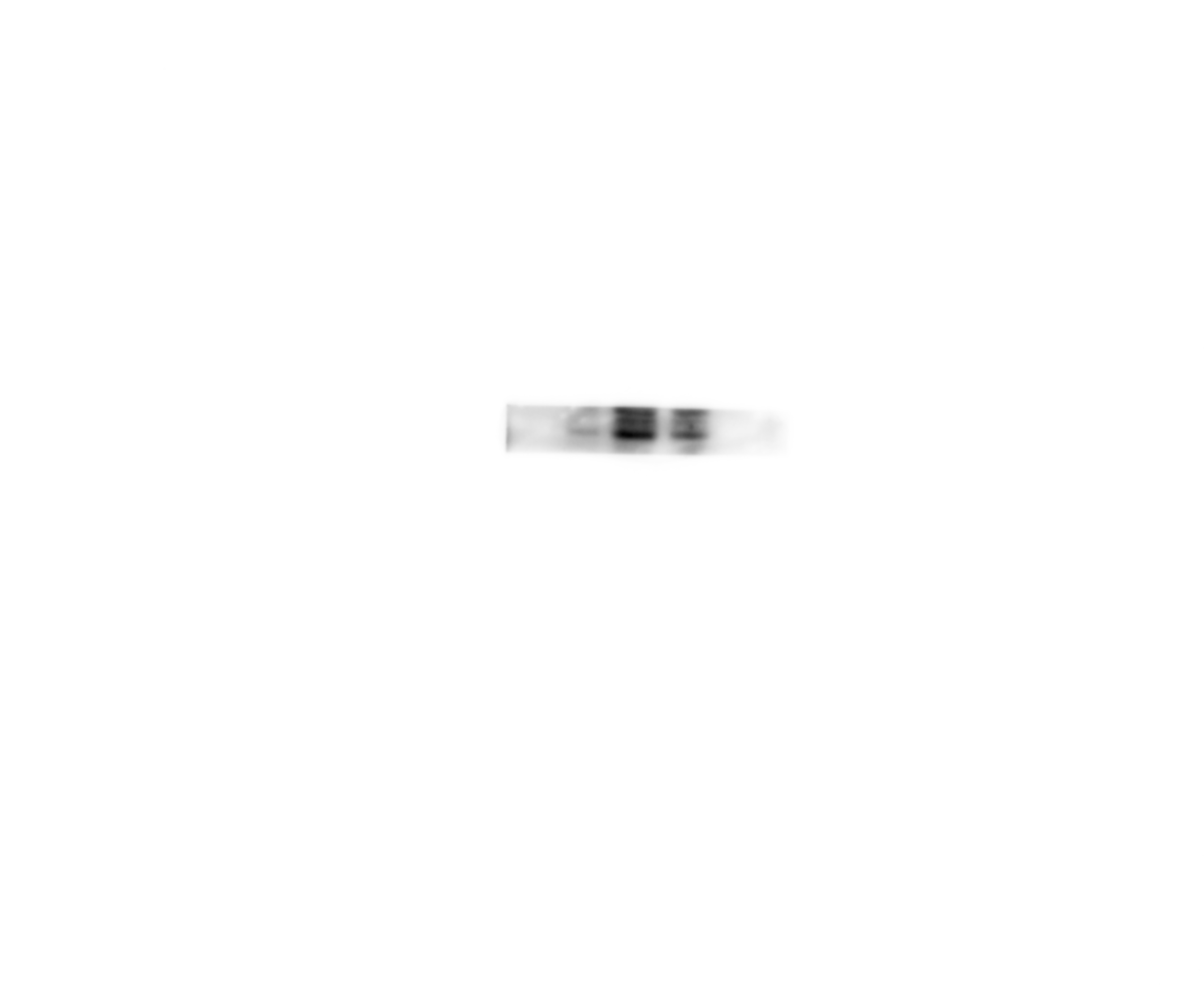

Supplement: Supplemental Information 5 [file peerj-12-17263-s005.zip › Figure 5 wb/002-shine[caspase3(2-2-2)0611].jpg]

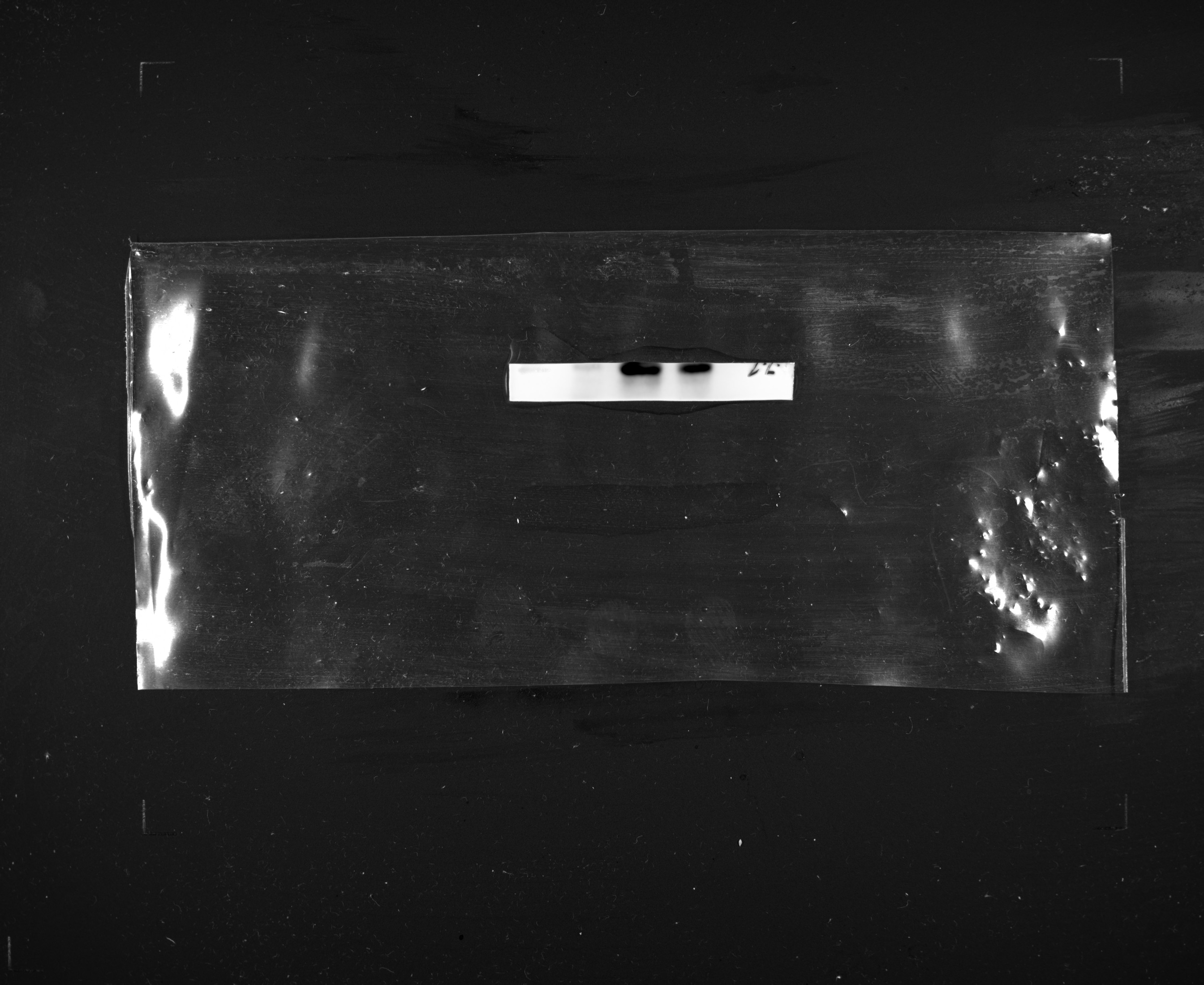

Supplement: Supplemental Information 5 [file peerj-12-17263-s005.zip › Figure 5 wb/003-merger[caspase9(2-2-1)0611].jpg]

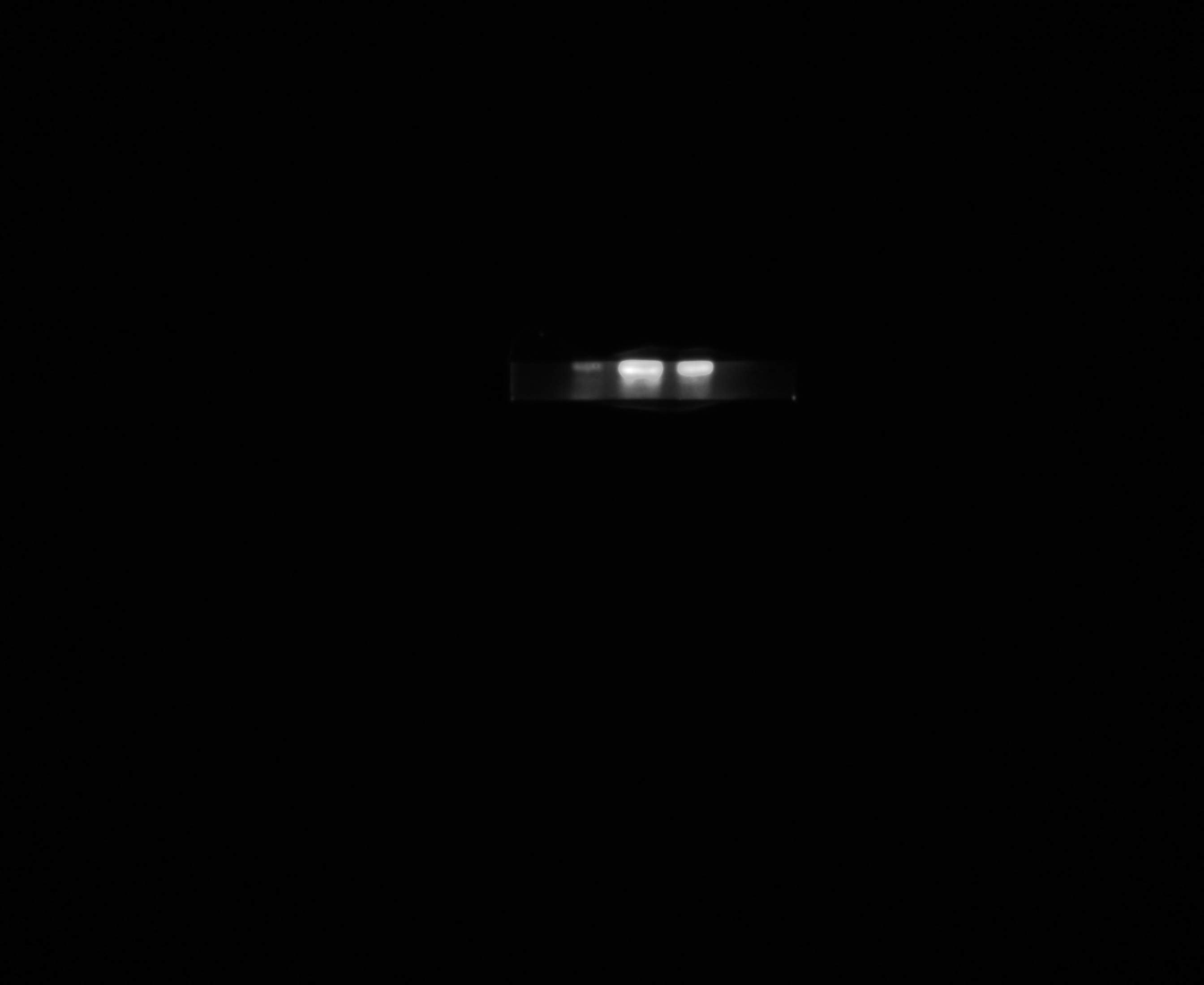

Supplement: Supplemental Information 5 [file peerj-12-17263-s005.zip › Figure 5 wb/003-shine[caspase9(2-2-1)0611]-raw[368,11124].tif]

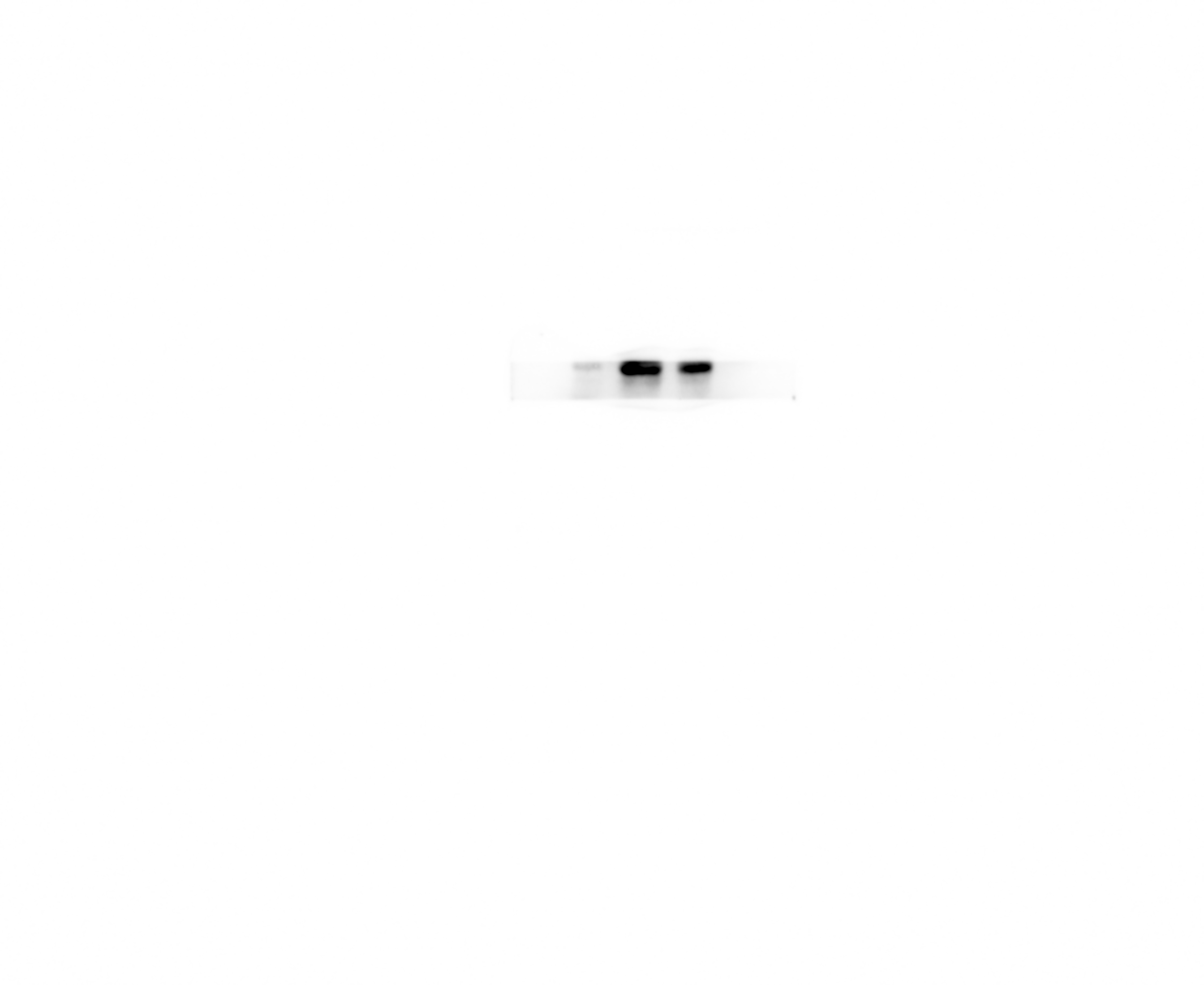

Supplement: Supplemental Information 5 [file peerj-12-17263-s005.zip › Figure 5 wb/003-shine[caspase9(2-2-1)0611].jpg]

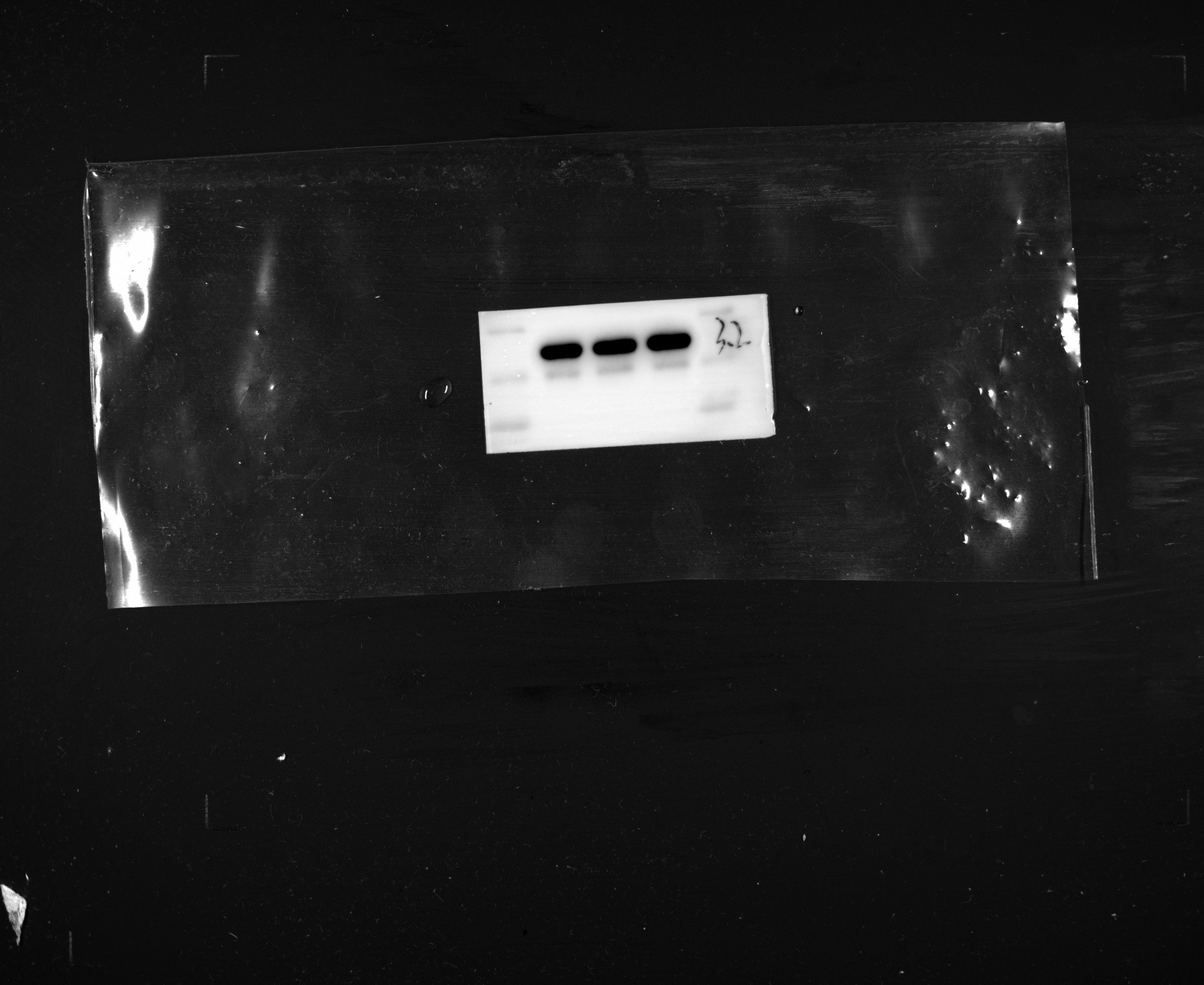

Supplement: Supplemental Information 5 [file peerj-12-17263-s005.zip › Figure 5 wb/004-merger[GAPDH3-2)0611].jpg]

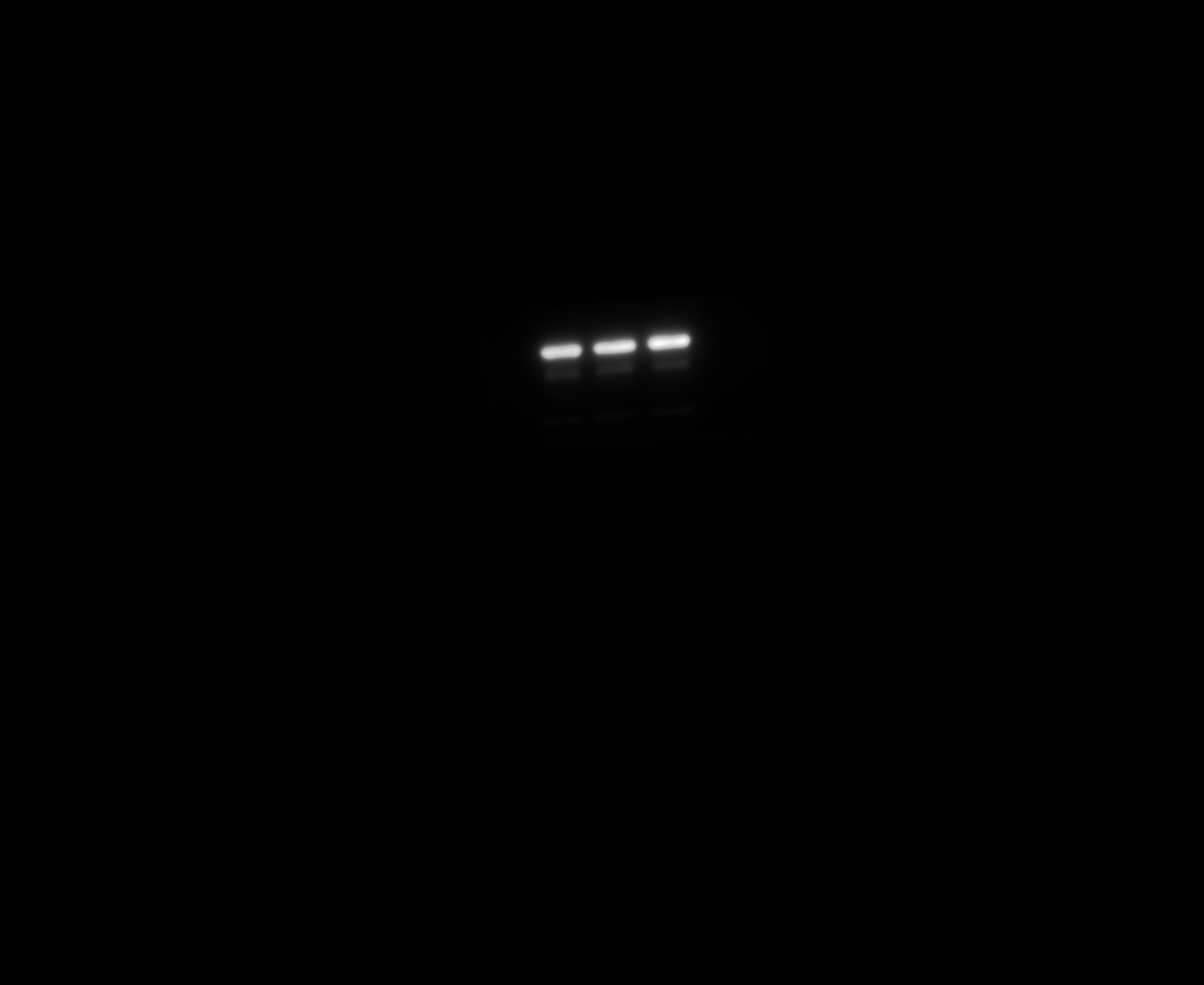

Supplement: Supplemental Information 5 [file peerj-12-17263-s005.zip › Figure 5 wb/004-shine[GAPDH3-2)0611]-raw[368,6015].tif]

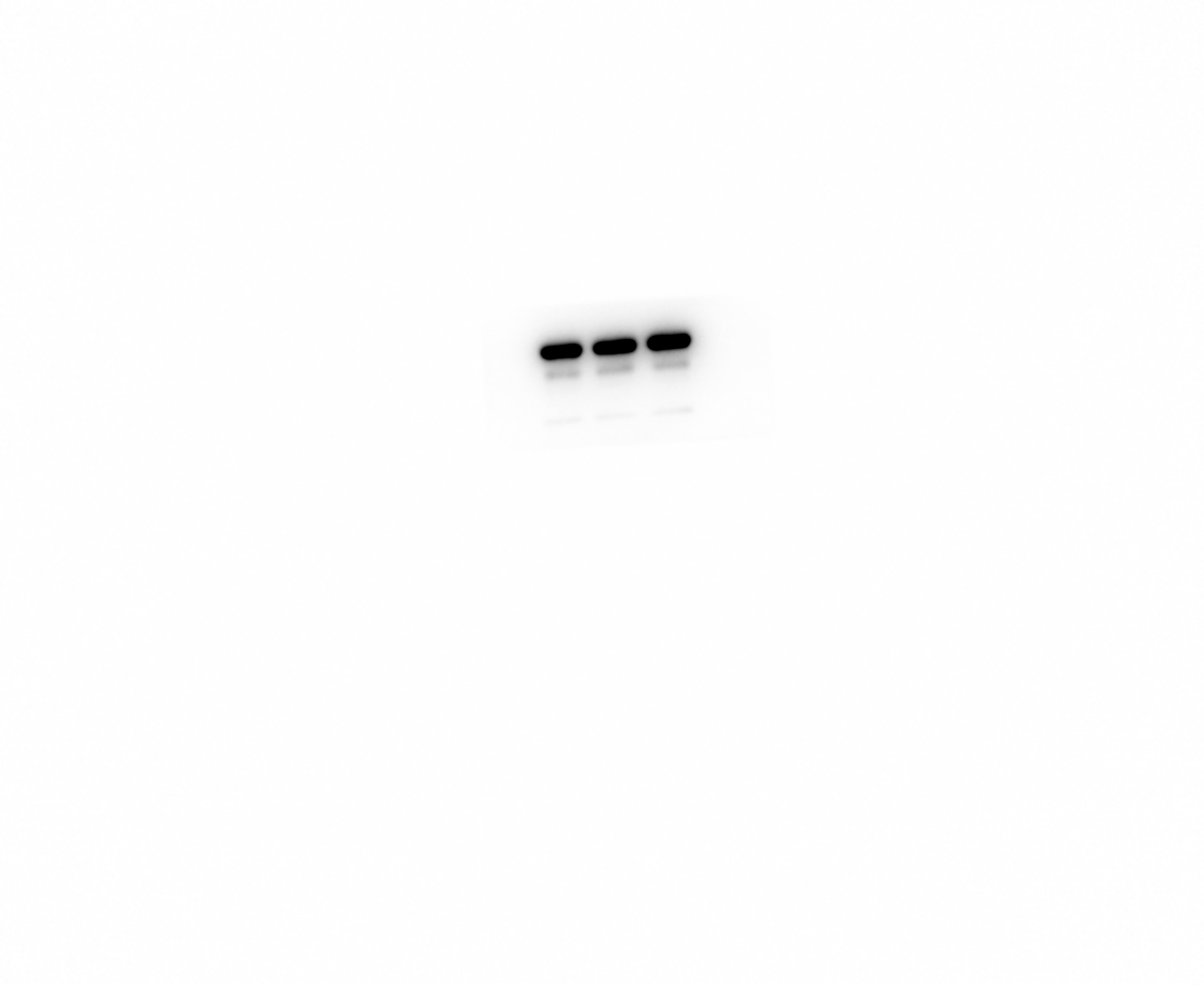

Supplement: Supplemental Information 5 [file peerj-12-17263-s005.zip › Figure 5 wb/004-shine[GAPDH3-2)0611].jpg]
